# Anti-inflammatory and cytotoxic assessment of flavonoids isolated from *Viola odorata* flowers with computer-guided docking study

**DOI:** 10.1038/s41598-025-19442-4

**Published:** 2025-09-18

**Authors:** Amal M. El-Feky, Ahmed A. El-Rashedy

**Affiliations:** 1 Pharmacognosy Department, National Research Centre, 33 El Bohouth St. (Former El Tahrir St.), Dokki, P.O. 12622, Giza, Egypt; 2 Chemistry of Natural and Microbial Products Department, National Research Center (NRC), Giza, Egypt; 3 Department Organic and Medicinal Chemistry, Faculty of Pharmacy, University of Sadat City, Menoufia, 32897 Egypt

**Keywords:** *V. odorata* flowers, Flavonoids, Anthocyanins, Phenolic acids, Anti-inflammatory, Anticancer, Biochemistry, Biotechnology, Cancer, Computational biology and bioinformatics, Drug discovery, Plant sciences, Health care, Medical research, Molecular medicine

## Abstract

The therapeutic potential of plant-derived polyphenols has garnered significant attention due to their roles in modulating inflammation and cancer progression. *Viola odorata* (*V. odorata*), traditionally used in herbal medicine, is known for its bioactive constituents, yet comprehensive profiling of its floral phytochemicals—particularly phenolic acids, flavonoids, and anthocyanins—remains limited. This study was pharmacologically designed to investigate the detailed phytochemical composition of *V. odorata* flowers and evaluate their anti-inflammatory and anticancer activities, aiming to identify novel natural agents for therapeutic development. Methanolic extracts of *V. odorata* flowers were analyzed for total phenolics, flavonoids, and anthocyanins using spectrophotometric methods. HPLC and UPLC/ESI–MS techniques were employed to identify and quantify individual compounds. Structural elucidation of isolated flavonoids was performed using advanced spectroscopic techniques. The anti-inflammatory and cytotoxic effects of the extract and isolated flavonoids were assessed in vitro using hepatocellular carcinoma (HepG2), human colonic epithelial (Caco-2), and colorectal carcinoma (HTC-116) cell lines. Molecular dynamics simulations were conducted to explore the interactions underlying the observed bioactivities. The methanolic extract contained 81.34 ± 0.17 mg GAE/g of total phenolics, 69.45 ± 0.24 mg CE/g of flavonoids, and 92.43 mg cyanidin-3-glucoside/100 g of anthocyanins. HPLC analysis revealed twelve phenolic acids and ten flavonoids, with gentisic acid (391.37 μg/g), apigenin-7-glucoside (417.22 μg/g), catechin (372.56 μg/g), and rutin (262.73 μg/g) being predominant. UPLC/ESI–MS identified 8 phenolic acids and derivatives, 3 flavonols, 4 flavones, 14 flavonoid glycosides, and 5 anthocyanins derived from cyanidin, delphinidin, and petunidin. Three flavonoids—5,7-dihydroxy-3,6-dimethoxyflavone, luteolin 7-*O*-glucoside, and kaempferol 3-*O*-rutinoside—were isolated and structurally characterized. The extract and isolated compounds exhibited dose-dependent cytotoxicity and anti-inflammatory effects across all tested cell lines. Molecular dynamics studies supported the observed inhibitory mechanisms. This study highlights the rich polyphenolic profile of *V. odorata* flowers and their promising anti-inflammatory and anticancer properties. The correlation between phytochemical composition and pharmacological activity underscores the therapeutic potential of *V. odorata* as a source of bioactive compounds for drug development.

**Supplementary Information:**

The online version contains supplementary material available at 10.1038/s41598-025-19442-4.

## Introduction

*Viola odorata*, also referred to as Banafshag and sweet violet, holds great importance within the Violaceae family. This plant is indigenous to Mediterranean countries and Asia Minor, but it has now expanded its presence to encompass a majority of Europe^1^. Its distinctive purple flowers, accompanied by dark green heart-shaped serrated leaves, thrive naturally in sunny climates found in tropical regions, often seen growing alongside hedges and along river banks^2^.

The fragrance of flowers has made them a popular ingredient in various perfuming products within the cosmetic industry^3^. From a scientific perspective, Banafshag flowers are recognized for their abundance of phyto-constituents such as flavonoids, and anthocyanins^4^. In a comprehensive analysis conducted by Batiha et al.^5^, it was revealed that *V. odorata* blossoms encompass approximately 1.1% flavonoids, 4% anthocyanins, 18% mucilage, and 8.5% ash. And upon investigating the effects of polyphenols, numerous studies have demonstrated the remarkable nutritional and health advantages offered by these compounds for human consumption^6^. Furthermore, the presence of phenolic and flavonoid compounds in these flowers has been closely linked to their ability to effectively eliminate harmful free radicals. This is achieved through the inhibition or modification of antioxidant enzymes and the binding of metal ions^7^.

In traditional medicines, the plant has been well-documented in treating a range of human ailments, including coughs, sore throat, hoarseness, and tonsillitis^1^. Numerous pharmacological studies have further explored the therapeutic features of different parts of *V. odorata*, including its leaves, flowers, aerial parts, and roots, in relation to the digestive, pulmonary, and nervous systems. These studies have highlighted its effectiveness as laxative^8^, anti-inflammatory^9^, sedative, pain reliever, as well as a sedative and hypotensive agent^1,10^. Additionally, Khan et al.^11^ found that the aqueous extract of *V. odorata* flowers exhibited antibacterial properties against *B. subtilis*, *E. coli*, and *S. aureus*. Furthermore, the entire aerial parts of the plant has been utilized in the treatment of bronchitis, cancer, cough, fever, urinary infections, rheumatism, sneezing, kidney and liver disorders^10^. Notably, Alipanah et al.^12^ reported that the whole aerial parts of *V. odorata*, including the stem, flowers, and leaves; possess significant cytotoxic effects on lung and liver cells, as well as antioxidant activity.

At present, researchers across the globe are increasingly intrigued by the potential of plant-based medications in preventing and treating various diseases. This surge in interest stems from the safety, affordability, effectiveness, and absence of adverse effects associated with these natural remedies, as opposed to synthetic medications. Nonetheless, the utilization of herbal drugs remains limited due to the lack of sufficient authentication^13^. Fortunately, modern scientific advancements have allowed us to bridge this knowledge gap by establishing connections between the pharmacological properties of herbal drugs and their active components.

Despite the wide use of *Viola odorata* in traditional medicine and the pharmacological evaluation of its various parts, the flowers specifically remain underexplored in terms of their complete phytochemical composition, particularly regarding phenolic acids, flavonoids, and anthocyanins—compounds known for their potent antioxidant, anti-inflammatory, and anticancer activities^4,5^. While previous studies have reported general therapeutic effects of *V. odorata*, there is a lack of detailed profiling and bioactivity assessment of its floral constituents. Given the high concentration of polyphenols in the flowers and their traditional use in treating inflammatory conditions, this study was designed to systematically investigate the flavonoid and anthocyanin composition of *V. odorata* flowers and evaluate their anti-inflammatory and anticancer potential using in vitro cell line models. By correlating the phytochemical profile with pharmacological activity, this research aims to fill a critical gap in the literature and support the development of flower-derived therapeutic agents.

### Chemicals

The requirements for High-Performance Liquid Chromatography (HPLC) and all compounds tested were of the utmost analytical purity. The phenolic compounds and flavonoids utilized in this investigation were sourced from Sigma-Aldrich Co., USA with ≥ 98% purity. COX-1 and COX-2 (ovine/human), as well as the 5-LOX enzyme (human recombinant) were obtained from Sigma-Aldrich. Dulbecco’s Modified Eagle’s Medium (DMEM), along with penicillin/streptomycin and L-glutamine, were acquired from Gibco BRL, California, USA. Fetal bovine serum (FBS) and 3-(4, 5-dimethylthiazol-2-yl)-2, 5-diphenylthiazolium bromide (MTT) were supplied by Sigma-Aldrich, St. Louis, Missouri, USA. The cancer cell lines used in this study, which include hepatocellular carcinoma (HepG2), human colonic epithelial cells (Caco-2), and human colorectal carcinoma cells (HTC-116), were obtained from the NAWAH scientific research center.

### Cell culture management

The assessment of cytotoxicity for the methanolic extract and isolated flavonoids was performed under controlled and sterile conditions within a Class II A2 Laminar flow biosafety cabinet. The carcinoma cell lines used in this study was obtained from Nawah Scientific Inc. (Cairo, Egypt)**.** The cells were cultured in Dulbecco’s Modified Eagle’s Medium, enriched with 10% heat-inactivated fetal bovine serum, 100 U/ml penicillin, and 100 μg/ml streptomycin sulfate, maintained at room temperature in humidified incubators with 5% CO_2_. Upon reaching approximately 80% confluence, the cells were subcultured using a trypsin–EDTA solution. Harvesting occurred during the logarithmic growth phase. The methanolic extract and isolated flavonoids were dissolved in DMSO and stored at − 20 °C. For each experimental trial, the stock solutions were mixed with the culture medium to obtain the required final concentrations^14^.

### Plant collection, authentication and extraction

*Viola odorata* flowers were procured from Giza’s Orman botanical garden during hosting Egypt’s Spring Flower Fair in March 2024 that organized by the Egyptian Ministry of Agriculture. The collection site is located at geographical coordinates 30.02917°N, 31.21306°E. The authentication of the specimen was graciously conducted by Mrs. Tereez Labib, Consultant of Plant Taxonomy at the Orman botanical garden in Giza, Egypt. A specimen has been archived in the herbarium of the National Research Centre (NRC) in Cairo, Egypt, identified by Voucher No. M228.

*V. odorata*’s fresh flowers were subjected to the process of air-drying, followed by crushing them into a fine powder. Afterwards, a meticulous extraction was performed on 250 g of the dried flower powder using methyl alcohol (10 times, 2 L for 40 days) following the methodology outlined by El Sawi et al.^15^. The resulting methanolic extract was subjected to evaporation under reduced pressure at 50 °C using rotatory evaporator (Heidolph, Germany), resulting in the formation of dark violet semi-solid residue weighing 38 g. Supplementary Figure S1 illustrates the extraction steps of *Viola odorata* flower.

### Quantification of total phenolics, flavonoids and anthocyanins

The quantification of total phenolics, total flavonoids, and total anthocyanins was performed using different techniques. The total phenolic content was determined using the Folin-Ciocalteu method, as described by El-Feky et al.^16^. The results were expressed as milligrams of gallic acid equivalent (mg GAE) per gram of extract. On the other hand, the total flavonoid content was estimated using the aluminum chloride methodology, as informed by Baba et al.^17^. The quantification was expressed as catechin equivalent (mg CE/g extract). Lastly, the total anthocyanins content was calculated by measuring the absorbance at different pH levels, following the procedure described by Lee et al.^18^, and the results were expressed as cyanidin-3-glucoside equivalents.

### Polyphenolic profiling

The extract *V. odorata* flowers was subjected to analysis using the High Pressure Liquid Chromatography (HPLC) technique by Agilent Technologies 1100 series liquid chromatography equipped with an autosampler and a Diode-Array Detector (DAD). The analytical column was an Eclipse XDB-C18 (150 × 4.6 μm; 5 μm) with a C18 guard column (Phenomenex, Torrance, CA). The mobile phase consisted of acetonitrile (solvent A) and 2% acetic acid in water (v/v) (solvent B). The gradient was programmed as follows: 0–5 min, 100% B (isocratic step); 30 min, 100–85% B (linear gradient); 20 min, 85–50% B (lin ear gradient); 5 min, 50–0% B (linear gradient); 5 min, 0 100% B (linear gradient) at a flow rate of 0.8 ml min^−1^, in order to detect various phenolic acids and flavonoids present in the methanolic extract. The identification of these compounds was accomplished by comparing their retention times and UV absorbance with authentic standards (Sigma-Aldrich, ≥ 98% purity, Shimadzu-UFLC Prominence) under identical chromatographic conditions^19^.

Furthermore, the analysis of flavonoids and anthocyanins was carried out using UPLC/ESI–MS analysis in negative and positive ion modes on a XEVO TQD triple quadruple instrument with Acquity UPLC—BEH C18 column (2.1 mm × 50 mm; 1.7 μm particle size), flow rate of 0.2 ml/min was maintained. The mobile phase consists of a binary solvent system of 0.1% aqueous formic acid (A) and acidified methanol in 0.1% formic acid (B). A gradient elution program was used for ESI–MS starting at 10% B, with a linear gradient to 30% B over 5 min, 70% B over 15 min, to 90% B over 22 min followed by isocratic of 90% B for 3 min, linear gradient to 100% (25–26 min). Returning to the isocriatic elution at 100% for 3min then quickly shifted to decline gradient elution to 10% B for 3min. Running temperature at room temp, source temperature 150 °C, cone voltage 30 eV, capillary voltage 3 kV, desolvation temperature 400 and 440 °C (+ ve and –ve mode, respectively), cone gas flow 50 L/h, and desolvation gas flow 780 and 900 L/h (+ ve and –ve mode, respectively); a mass spectrometer manufactured by Waters Corporation located in Milford, MA 01,757 USA. The ACQUITY UPLC-BEH with C18 as column 1.7 μm, 2.1 × 50 mm was utilized for the analysis as described by Paunova-Krasteva et al.^20^.

### Isolation and identification of major flavonoids

The methanolic extract of *V. odorata* flowers (5 g) was subjected to silica gel column chromatography (120 × 2.5 cm) with elution commencing from chloroform and gradually increasing the polarity with methanol. Fractions of 50 ml were concentrated under reduced pressure and examined on TLC plates using chloroform – methanol (9:1, v/v) developing system. Fractions with similar characteristics were combined together.

Fraction of chloroform–methanol (95:5) was purified on TLC of silica gel GF254 (20 × 20 cm, with a thickness of 0.25mm, MERCK) using n-hexane/EtOAc (4:1 v/v) as the mobile phase^21^. This purification process resulted in a pure spot that exhibited a yellow color upon exposure to ammonia vapor and AlCl_3_ spray reagent (compound 1). While fraction of chloroform–methanol (80:20) was further subjected to silica gel column chromatography (25 × 1.3 cm) using different ratios of chloroform and ethyl acetate as the eluting system. This process resulted in the formation of two main subfractions, SF1 (75:25 v/v) and SF2 (65:35 v/v). To purify SF1 and SF2, TLC silica gel GF254 (20 × 20 cm) was employed using a developing system consisting of n-hexane–ethyl acetate–methanol-water (5:5:5:5, v/v)^22^. The TLC purification revealed the presence of two spots that exhibited positive responses to flavonoid spray reagents, indicating the presence of compounds 2 and 3, respectively. The identification of these pure isolated compounds was accomplished through determination of their melting points and by different spectroscopic techniques. Additionally, relevant literature was reviewed to further confirm their identities. Supplementary Figure S2 illustrates the chromatographic separation, and identification of major flavonoids from *Viola odorata* flower methanolic extract.

### Anti-inflammatory evaluation

The methanolic extract of *V. odorata* flowers and isolated flavonoids were subjected to an in vitro anti-inflammatory assessment by inhibiting two isoenzymes, cyclooxygenase COX-1 and COX-2 (ovine/human), as well as the 5-LOX enzyme (human recombinant). The inhibition assays for COX-1 and COX-2 were conducted using the COX-1 and COX-2 kit (Cayman, No.: 560131) as described by Alaa et al.^23^. The 5-lipoxygenase inhibition assay was performed with the 5-LOX kit (No. 437996, Sigma-Aldrich) following the method outlined by Huang et al.^24^, and the IC_50_ value was determined through linear regression analysis according to El Feky & El-Rashedy^25^.

### Cytotoxicity evaluation

The cytotoxic assessment of *V. odorata* flower extract and its isolated flavonoids was performed against three human cell lines: hepatocellular carcinoma (HepG2), human colonic epithelial cells (Caco-2), and human colorectal carcinoma cells (HTC-116) utilizing the MTT assay as outlined by Mosmann^26^. The cells were treated with serial dilutions of the extract and isolated compounds over a period of forty-eight hours, with final concentrations of 100, 50, 25, 12.5, and 6.25 µg/ml. The effectiveness of the extract was compared to that of the reference drug, Doxorubicin. The percentage of cell viability was calculated by taking the ratio of the absorbance of the plant extract to that of the control, multiplied by 100. Furthermore, the IC_50_ value of the plant extract was determined using Sigma Plot software version 11 (Spw.exe) (https://sigmaplot.software.informer.com/11.0/).

### System preparation and molecular docking

The crystal structures of Human Cyclooxygenase-2 (COX-2) and Vascular Endothelial Growth Factor Receptor 1 (VEGFR-1) were sourced from the Protein Data Bank, designated by codes 3LN1^27^ and 3HNG, respectively. Preparation of the structures was conducted using UCSF Chimera^28^. The pH was adjusted and optimized to 7.5 through the use of PROPKA^29^. The two-dimensional structure was created with ChemBioDraw Ultra 12.1^30^. The steepest descent method and the MMFF94 force field were utilized in Avogadro software^31^ for the purpose of energy minimization. Before proceeding with the docking process, hydrogen atoms were removed using UCSF Chimera^28^.

### Molecular docking

Docking calculations were conducted utilizing AutoDock Vina^32^, with Gasteiger partial charges^33^ assigned during the docking process. The AutoDock graphical user interface from MGL tools was employed to define the AutoDock atom types^34^. The coordinates for the AutoDock Vina grid center were set at 32.12, − 14.58, − 21.93 (3LN1), and 5.175, 20.19, 26.83(3HNG), while the dimensions of the search space were configured to 20 Å × 20 Å × 20 Å, with an exhaustiveness parameter of 8. The Lamarckian genetic algorithm^35^ was applied to produce docked conformations in a descending order based on docking energy.

### Molecular dynamic simulations

The utilization of Molecular Dynamics (MD) simulations in the examination of biological systems facilitates the investigation of atomic and molecular movements that are challenging to observe through alternative methods^36^. The knowledge acquired from these simulations offers an in-depth perspective on the dynamic alterations occurring within biological systems, including changes in conformation and molecular interactions^36^. All MD simulations for the respective systems were conducted using the GPU variant of the PMEMD engine available in the AMBER 18 software suite^37^. The calculation of the partial atomic charge for each compound was performed using the General Amber Force Field (GAFF) method as implemented in ANTECHAMBER^38^. Each system was implicitly solvated by the Leap module of the AMBER 18 software, which utilized an orthorhombic box filled with TIP3P water molecules, ensuring that the distance from any box edge was maintained at 10 Å. The Leap module facilitated the neutralization of each system through the introduction of Na + and Cl − counter ions. Initially, a 2000-step minimization process was conducted for each system, employing a restraint potential of 500 kcal/mol. This was succeeded by a 1000-step complete minimization utilizing the conjugate gradient algorithm, executed without any restraints.

In the course of the molecular dynamics simulation, each system underwent a gradual temperature increase from 0 to 300K over a duration of 500 picoseconds, ensuring uniformity in the number of atoms and volume across all systems. The solutes within the systems were subjected to a harmonic potential constraint of 10 kcal/mol, accompanied by a collision frequency of 1 picosecond. Subsequently, each system was further heated and equilibrated at a constant temperature of 300K for an additional 500 picoseconds. To achieve an isobaric-isothermal (NPT) ensemble, the number of atoms and the pressure in each system were held constant during the production simulations, with the pressure regulated at 1 bar through the application of the Berendsen barostat^39^.

Each system underwent molecular dynamics simulations. The SHAKE algorithm was employed to maintain the constraints on hydrogen bond atoms throughout the simulations. A time step of 2 femtoseconds was utilized in each simulation, which incorporated a single precision floating-point (SPFP) model. The simulations were conducted under an isobaric-isothermal ensemble (NPT) framework, featuring randomized initial conditions, a constant pressure of 1 bar, a pressure coupling constant of 2 picoseconds, a temperature set at 300 K, and a Langevin thermostat operating with a collision frequency of 1 picosecond.

### Post-MD analysis

Following the collection of trajectories from molecular dynamics simulations at 1 ps intervals, an analysis was conducted using the CPPTRAJ module within the AMBER18 suite^40^. The graphical representations and visualizations were generated using the Origin data analysis software^41^ alongside Chimera^28^.

### Thermodynamic calculation

The Poisson-Boltzmann and generalized Born models, along with surface area continuum solvation methods (MM/PBSA and MM/GBSA), have proven effective for estimating ligand-binding affinities^42^. These methodologies utilize molecular simulations of protein–ligand complexes to calculate the binding free energy through a defined force field. The binding free energy is averaged over 200 snapshots taken from a comprehensive 20 ns trajectory. According to Hou et al.^43^, the change in binding free energy (ΔG) for each molecular entity, including the complex, ligand, and receptor, can be accurately assessed. The variables Egas, Eint, Eele, and Evdw represent the gas-phase energy, internal energy, Coulomb energy, and van der Waals energy, respectively. The gas-phase energy (Egas) was determined directly from the parameters of the FF14SB force field. The solvation-free energy (Gsol) was calculated by considering the contributions from both polar states (GGB) and non-polar states (G). The non-polar solvation free energy (GSA) was derived from the Solvent Accessible Surface Area (SASA) methodology, utilizing a water probe radius of 1.4 Å, as outlined by Greenidge et al.^44^. Conversely, the polar solvation contribution (GGB) was evaluated through the application of the GB equation. The symbols S and T denote the total entropy of the solute and the temperature, respectively. The MM/GBSA-binding free energy approach implemented in Amber18 was employed to assess the contribution of each residue to the overall binding free energy.

### Statistics

The statistical evaluation of the parameters under investigation was carried out using SPSS 9.05 (USA). Data are presented as mean ± SE from three replicates. Significant variations were analyzed using one-way analysis of variance (ANOVA), subsequently followed by the Co-state computer program (SPSS for Windows, version 11.0).

## Results

### Total phenolics, flavonoids and anthocyanins

The concentrations of total phenolic and flavonoid compounds in the methanolic extract of *V. odorata* flowers were determined to be 81.34 ± 0.17 (mgGAE/g) and 69.45 ± 0.24 (mg CE/g), respectively**.** Additionally, the anthocyanin content in the extract was measured to be 92.43 mg of cyaniding-3-glucoside per 100g of extract. These findings support the rich polyphenolic profile of *V. odorata*, which aligns with its traditional use in inflammation-related disorders. The high anthocyanin content suggests potential antioxidant benefits, possibly contributing to the observed pharmacological activities.

### HPLC analysis of phenolic acids and flavonoids

The identification of phenolic acids and flavonoid constituents in the methanolic extract of *V. odorata* flowers was carried out using high pressure liquid chromatography (HPLC), as detailed in Table 1. A total of twelve phenolic acids and ten flavonoids were detected in the extract. Among these compounds, gentisic acid was found to be the most abundant, with a concentration of 391.37 μg/g. The prominent flavonoids identified were apigenin-7-glucoside (417.22 μg/g), catechin (372.56 μg/g), and rutin (262.73 μg/g). These compounds are well-documented for their anti-inflammatory and anticancer potential. HPLC chromatogram of phenolic acids and flavonoids in the methanolic extract of *V. odorata* flowers at 280, 320, and 360 nm was illustrated in Supplementary Figure S3.

**Table 1:** HPLC analysis of phenolic acids and flavonoids in the methanolic extract of *V. odorata* flowers.

No	Identified compounds	R_*t*_	λmax /nm	Concentration (μg/g)
1	Gallic acid	4.757	280	293.74
2	Protocatechuic acid	6.911	280	109.31
3	*p*- Hydroxybenzoic acid	10.284	280	354.28
4	Gentisic acid	12.440	280	391.37
5	Apigenin	12.444	360	107.64
6	Catechin	13.942	320	372.56
7	Chlorogenic acid	14.993	280	253.96
8	Caffeic acid	16.939	320	198.68
9	Naringenin	18.274	280	93.95
10	Syringic acid	20.456	280	182.68
11	Vanillic acid	21.275	280	118.69
12	Ferulic acid	21.957	320	276.53
13	Sinapic acid	23.648	320	192.17
14	*p*-coumaric acid	27.600	280	234.35
15	Rutin	29.528	360	262.73
16	Hesperidin	29.119	280	96.35
17	Naringin	30.105	280	104.18
18	Cinnamic acid	35.785	280	92.64
19	Apigenin-7-glucoside	36.370	360	417.22
20	Quercetin	40.085	360	139.41
21	Kaempferol	56.641	360	221.73
22	Chrysin	57.431	360	248.94

### UPLC/ESI–MS analysis of flavonoids and anthocyanins

To identify the phenolic acids, flavonoids, and anthocyanins present in *V. odorata* flowers, LC–ESI–MS negative and positive ionization techniques were employed. Through analysis of molecular weights, mass fragmentation patterns, and a thorough review of previous literature, a total of 34 compounds were successfully identified and documented in Table 2. Supplementary Figure S4 shows UPLC/ESI–MS chromatogram of the methanolic extract of *V. odorata* flowers in negative (A) and positive ionization mode (B).

**Table 2:** Identification of phenolic acids, flavonoids and anthocyanins in the methanolic extract of *V. odorata* flowers by UPLC/ESI–MS analysis (negative and positive ionization mode).

No	Parent ion *m/z*		MS/MS fragments (m/z)	Tentative identification	Molecular Formula	Chemical class	References
	[M-H]^-^ (R_*t*_ , min)	[M + H]^+^ (R_*t*_, min)					
1	169.1082 (1.09)		151[M-H-H_2_O], 125[M-H-CO_2_]	Gallic acid	C_7_H_6_O_5_	Phenolic acid	^45^
2	153.0350 (1.18)		135[M-H-H_2_O], 109[M-H-CO_2_], 112	Protocatechuic acid	C_7_H_6_O_4_	Phenolic acid	^45^
3	179.0230 (1.34)		161 [M-H-H_2_O], 135[M-H-CO_2_],101, 89	Caffeic acid	C_9_H_8_O_4_	Phenolic acid	^45^
4	163.0754 (2.06)		145[M-H-H_2_O], 119[M-H-CO_2_]	*P*-Coumaric acid	C_9_H_8_O	Phenolic acid	^45^
5	193.1161 (5.46)		178[M-H-CH_3_],175[M-H-H_2_O], 149[M-H-CO_2_], 134[M-H-CH_3_-CO_2_]	Ferulic acid	C_10_H_10_O_4_	Phenolic acid	^45^
6	337.0916 (5.77)		319[M-H-H_2_O], 293[M-H-CO_2_], 175[M-H-coumaroyl]	*P*-Coumaroylquinic acid	C_16_H_18_O_8_	Phenolic acid derivative	^45^
7	367.2271 (7.24)		349[M-H-H_2_O], 323[M-H-CO_2_], 191[M-H-feruloyl (176)], 173[M-H-feruloyl- H_2_O]	Feruloylquinic acid	C_17_H_20_O_9_	Phenolic acid derivative	^45^
8	341.1964 (11.25)		179[M-H-caffoeyl], 161[M-H-caffoeyl-H_2_O],135[M-H-caffoeyl-CO_2_]	Caffeoyl hexoside	C_15_H_18_O_9_	Phenolic acid derivative	^45^
9	301.0717 (11.41)	303 (11.45)	273[M-H–CO], 255[M-H–CO-H_2_O], 179, 151	Quercetin	C_15_H_10_O_7_	Flavonoid flavonol	^46^
10		904.2611 (11.78)	742 [M + H-Coum], 434 [M + H-Coum-Rut] , 287 [Cyanidin]	Cyanidin-3-(4´´-*p*-coumaroyl)-rutinoside-5-glucoside	C_42_H_47_O_22_	Anthocyanin	^47^
11		611.1838 (12.39)	465[M + H-Rham],449 [M + H-Glu], 303 [M + H-Rut], 179,153	Hesperidin (hesperetin 7-O- rutinoside)	C_28_H_34_O_15_	Flavonoid glycoside	^13^
12		920.3356 (12.75)	758 [M + H-Coum], 450 [M + H-Coum-Rut], 303[Delphinidin]	Delphinidin-3-(4´´-*p*-coumaroyl)-rutinoside-5-glucoside	C_42_H_47_O_23_	Anthocyanin	^47^
13		389.3367 (12.78)	374[M + H-CH_3_], 359[M + H-CH_2_O], 341[M + H-3CH_4_], 227[RDA, ring B]	6-Hydroxy 3’,4’,5,7,8 pentamethoxy flavone	C_20_H_20_O_8_	Flavonoid flavone	^48^
14		287.243652 (12.87)	269[M + H-H_2_O], 231, 213, 171,165,153, 135	Luteolin	C_15_H_10_O_6_	Flavonoid flavone	^46^
15		343.1180 (14.06)	328[M + H-CH_3_], 313[M + H-CH_2_O], 299[M + H-CH_4_-CO], 283[M + H-4CH_3_], 282[M + H-2CH_2_O-H]	4’,5,6,7-tetramethoxy flavone	C_19_H_18_O_6_	Flavonoid flavone	^48^
16	431.2070 (15.58)		269[M-H-Glu], 227 , 179, 151	Apigenin 7-*O*-glucoside	C_21_H_20_O_10_	Flavonoid glycoside	^49^
17		433.2409 (15.87)	415[M + H-H_2_0], 313[M + H-120], 283 [M + H-150]	Apigenin 8-*C*-glucoside	C_21_H_20_O_10_	Flavonoid glycoside	^49^
18	417.2197 (15.91)		285[M-H-Arab], 255[M-H- Arab-CH_2_O], 227	Kaempferol 3-*O*- arabinoside	C_20_H_18_O_10_	Flavonoid glycoside	^49^
19		313.3206 (16.13)	298 [M + H-CH_3_], 297 [M + H-CH_4_], 270 [M + H-CH_3_-CO], 269 [M + H-CH_4_-CO], 255 [M + H-2CH_3_-CO]	5,7,4’-Trimethoxyflavone	C_18_H_16_O_5_	Flavonoid flavone	^48^
20	433.1585 (16.04)	435.3871 (16.25)	301[M-H-Arab], 271[M-H-Arab-CH_2_O], 255[M-H-Arab-CO-H_2_O]	Quercetin 3-*O*-arabinoside	C_20_H_18_O_11_	Flavonoid glycoside	^49^
21	317.1116 (17.09)		299[M-H-H_2_O] 289[M-H–CO], 273, 259[M-H-2CHO] 245, 179, 151	Myricetin	C_15_H_10_O_8_	Flavonoid flavonol	^13^
22	315.1950 (17.58)		300[M-H-CH_3_], 269,246, 179, 151	Isorhamnetin	C_16_H_12_O_7_	Flavonoid O- methylated flavonol	^46^
23	447.2761 (18.41)	449.3243 (18.43)	285[M-H-Glu], 255[M-H-Glu-CH_2_O], 227[M-H-Glu-2CHO]	Kaempferol 3-*O*- glucoside	C_21_H_20_O_11_	Flavonoid glycoside	^50^
24	463.1482 (18.78)	465.2608 (19.30)	301[M-H-Glu], 271[M-H-Glu-CH_2_O], 255[M-H-Glu-CO-H_2_O]	Quercetin 3-*O*-glucoside	C_21_H_19_O_12_	Flavonoid glycoside	^50^
25	447.2761 (18.98)		401[M-H-H_2_O-CO], 285[M-H-Glu], 267[M-H-Glu-H_2_O], 255,319,363	Luteolin 8-*O*- glucoside	C_21_H_20_O_11_	Flavonoid glycoside	^49^
26		480.2705 (19.83)	317[Petunidin], 303[M + H-Glu-CH_3_], 286[M + H-Glu-CH_3_OH], 272, 264, 229	Petunidin 3-*O*-glucoside	C_22_H_23_O_12_	Anthocyanin	^47^
27		595.2519 (19.84)	449[M + H-Rhm], 433[M + H-Glu], 287[M + H-Rut], 262, 145	Luteolin 7-*O*-rutinoside	C_27_H_30_O_15_	Flavonoid glycoside	^49^
28		481.2231 (19.98)	319[M + H-Glu], 273[M + H-Glu-CO-H_2_O], 245, 153	Myricetin 3-*O*-glucoside	C_21_H_20_O_13_	Flavonoid glycoside	^49^
29	577.4143 (19.33)	579.2617 (20.26)	269[M-H-Rut], 223[M-H-Rut-CO-H_2_O],179, 112	Apigenin -7-*O*-rutinoside	C_27_H_30_O_14_	Flavonoid glycoside	^50^
30		612.3564 (20.66)	450 [M + H-Coum], 303[Delphinidin], 288 [M + H-Coum-Glu],	Delphinidin-3-*O*-(6-*p*-coumaroyl) glucoside	C_30_H_27_O_14_	Anthocyanin	^47^
31	609.2702 (23.93)		301[M-H-Rut], 271[M-H-Rut-CH_2_O], 255[M-H-Rut-CO-H_2_O], 179, 151	Rutin	C_27_H_30_O_16_	Flavonoid glycoside	^46^
32	593.2776 (25.38)		285[M-H-Rut], 255[M-H-Rut-CH_2_O], 227[M-H-Rut-2CHO]	Kaempferol 3-*O*-rutinoside	C_27_H_30_O_15_	Flavonoid glycoside	^49^
33		625.2913 (26.42)	479[M + H-Rham], 317[M + H-Rut], 327	Isorhamnetin 3-*O*-rutinoside	C_28_H_32_O_16_	Flavonoid glycoside	^46^
34		612.1839 (27.03)	466[M + H-Rha], 450[M + H-Glu], 303[Delphinidin]	Delphinidin 3-*O*-rutinoside	C_27_H_31_O_16_	Anthocyanin	^47^

In the negative ionization mode, five phenolic acids and three derivatives were tentatively identified in *V. odorata* flowers. These phenolic acids and their derivatives (1–8) exhibited the same fragmentation pathway characterized by the loss of the CO_2_ group and water. Notably, ferulic acid (compound 5, R_t_ 5.46) with a molecular ion [M-H]^−^ 193 additionally displayed the loss of a methyl group, resulting in a fragment ion at *m/z* 178.

However, the profiling of flavonoids in *V. odorata* flowers was conducted using LC–ESI–MS in both negative and positive ionization modes. The analysis revealed the presence of various flavonoids, which were classified into different subclasses. In the flavonol subclass, three compounds were identified, namely quercetin, myricetin, and isorhamnetin. Additionally, four flavones were identified, namely luteolin, 4′,5,6,7-tetramethoxy flavone, 6-Hydroxy 3’,4’,5,7,8 pentamethoxy flavone, and 5,7,4’-Trimethoxyflavone. Furthermore, the analysis revealed the presence of fourteen flavonoid glycosides, including hesperetin 7-*O*-rutinoside, apigenine 7-*O*-glucoside, apigenin 8-*C*-glucoside, kaempferol 3-*O*-arabinoside, quercetin 3-*O*-arabinoside, kaempferol 3-*O*-glucoside, quercetin 3-*O*-glucoside, luteolin 8-*O*-glucoside, luteolin 7-*O*-rutinoside, myricetin 3-*O*-glucoside, apigenine 7-*O*-rutinoside, rutin, kaempferol 3-*O*-rutinoside, and isorhamnetin 3-*O*-rutinoside.

Through a meticulous analysis of the mass fragmentation pattern, three flavones were identified in the mass spectra of *V. odorata* . These compounds followed the RDA fragmentation pathway, with the [1,3A]^−^ and [1,2 A]^−^ fragments being the most commonly formed. The unsubstituted fragments were observed at *m/z* 179 and *m/z* 151, respectively**.** Additionally, the compounds exhibited loss of H_2_O and CO molecules**.** They could be detected in the fragmentation ways of compounds 9 and 21, compound 22 displayed a similar fragmentation pattern, with the formation of *m/z* 300 due to the loss of a methyl group.

In the positive mode, the methoxy flavones (compounds 13, 15, and 19) experienced fragmentation through methyl loss, resulting in the production of *m/z* 374, 328, and 298, respectively. The flavone compound 14 generated a fragment at *m/z* 269 due to the loss of water**.**

The identification of flavonoid glycosides was achieved in both positive and negative modes by cleaving the glycosidic *O*-linkages, resulting in the removal of monosaccharide residues accompanied by H-rearrangement. In the case of compounds 11, 27, 29, 31–33, the elimination of rutinoside led to the production of a fragment peak corresponding to the aglycone. On the other hand, compounds 16, 23–25, and 28 underwent mass fragmentation by removing glucoside, while compounds 18 and 20 eliminated rhamnoside**.**

In the present investigation, positive mode analysis revealed the identification of five anthocyanins. These include cyanidin 3-(4″-*p*-coumaroyl)-rutinoside-5-glucoside, delphinidin 3-(4″-*p*-coumaroyl)-rutinoside-5-glucoside, petunidin 3-*O*-glucoside, delphinidin 3-*O*-(6-*p*-coumaroyl) glucoside, and delphinidin 3-*O*-rutinoside. The anthocyanins derived from aglycones cyanidin, delphinidin, and petunidin were identified based on the presence of specific fragment signals at *m/z* 287, 303, and 317, respectively, in their mass fragmentation analysis**.** Violanin, also known as Delphinidin-3-(4″-*p*-coumaroyl)-rutinoside-5-glucoside, compound 12, exhibited a retention time of 12.75 min in the positive ion mode. Its characteristic molecular ion at *m/z* 920, corresponding to [M + H], and a peak at *m/z* 303, corresponding to the delphinidin aglycone, were observed. Additionally, the loss of coumaroyl and rutinosyl moieties resulted in the appearance of other characteristic ions at *m/z* 758 [M + H -Coum] and *m/z* 450 [M + H-Coum-Rut]. Notably, compound 10 (R_*t*_ 11.78 min), 26 (R_*t*_ 19.83 min), 30 (R_*t*_ 20.66 min), and 34 (R_*t*_ 27.03 min) exhibited similar mass fragmentation patterns of anthocyanins.

### Identification of the isolated flavonoids

Compound (1), *5,7-Dihydroxy-3,6-dimethoxyflavone* was obtained in the form of yellow crystals, which exhibited a melting point of 175 °C. R_*f*_ 0.73, the UV-λmax (MeOH) values were recorded at 258 nm and 335 nm, with an absorbance value below 350 nm, indicating the presence of a flavone nucleus. When treated with MeOH + NaOMe, bathochromic shifts were observed at 260 nm, 275 nm, and 339 nm, confirming the existence of polyhydroxyl groups. Furthermore, the addition of MeOH + AlCl_3_ resulted in bathochromic shifts at 278 nm, 305 nm (shoulder), 345 nm (shoulder), and 425 nm, which were attributed to the presence of a free hydroxyl group at C-5. The stability of UV absorbance in band II upon treatment with MeOH + AlCl_3_/HCl at 279 nm, 305 nm, and 395 nm further confirmed the presence of a free hydroxyl group at C-5. Additionally, when subjected to MeOH + NaOAc, bathochromic shifts were observed at 285 nm, 308 nm, and 396 nm, indicating the presence of a free 7-OH group. However, no change in UV absorbance was observed when treated with MeOH + NaOAc/H_3_BO_3_ at 283 nm and 395 nm, suggesting the absence of *ortho*-dihydroxyl groups. In the ^1^H-NMR spectrum (500 MHz, CD_3_OD), the chemical shifts (δ / ppm) were observed at 3.59 (3H, s, 6-OCH_3_), 3.79 (3H, s, 3-OCH_3_), 6.59 (1H, s, H-8), 7.42 (2H, d, H-3′, H-5′), 7.46 (1H, s, H-4′), 7.98 (2H, m, H-2′,H-6′), 11.34 (1H, s, 7-OH), 12.05 (1H, s, 5-OH). The ^13^C-NMR spectrum (125 MHz, CD_3_OD) showed chemical shifts (δ / ppm) at 59.02 (6-OCH_3_), 61.11 (3-OCH_3_), 95.78 (C-8), 103.46 (C-10), 128.18 (C-2′, C-6′), 128.78 (C-3′, C-5′),130.46 (C-6), 131.85 (C-4′),133.25 (C-1′), 139.92 (C-3), 148.23 (C-5), 149.02 (C-9), 153.23 (C-2), 156.18 (C-7), 174.43 (C-4).

Compound (2), *luteolin 7-O-glucoside* was obtained in the form of yellow crystals, which exhibited a melting point of 255 °C. R_*f*_ 0.85, the UV-λmax (MeOH) spectrum displayed absorption peaks at 255, 268, and 345 nm, with absorbance below 350 nm, indicating the presence of a flavone nucleus. When treated with MeOH + NaOMe, a bathochromic shift was observed at wavelengths 261, 315, and 390 nm, confirming the existence of polyhydroxyl groups. Similarly, the addition of MeOH + AlCl_3_ resulted in a bathochromic shift at 270, 300, and 435 nm, suggesting the presence of a hydroxyl group at C-5 and *ortho*-dihydroxyl groups. Further treatment with MeOH + AlCl_3_/HCl exhibited stable UV absorbance in band II at 269, 299, and 400 nm, indicating the presence of a hydroxyl group at C-5, while a hypsochromic shift in band I indicated the presence of a 3′, 4′-dihydroxy group. The addition of MeOH + NaOAc caused a bathochromic shift in band I at 256, 370, and 415 nm, which could be attributed to the presence of a free 4’-OH group. Finally, when treated with MeOH + NaOAc/H_3_BO_3_, a bathochromic shift in band I at 254, 385, and 420 nm indicated the presence of a 3′, 4′-dihydroxy group. In the ^1^H-NMR spectrum (500 MHz, CD_3_OD), the chemical shifts (δ) and corresponding proton signals were observed at 6.33 ppm (1H, d, J = 2.5Hz, H-6), 6.58 ppm (1H, d, J = 2.5Hz, H-8), 6.50 ppm (1H, s, H-3), 7.69 ppm (1H, d, J = 2.8Hz, H-2′), 6.90 ppm (1H, d, J = 8.2Hz, H-5′), 7.54 ppm (1H, dd, J = 2.8, 8.2Hz, H-6’), and 5.12 ppm (H-1′′). ^13^C-NMR (125 MHz, CD_3_OD) *δ* / ppm: 165.51 (C-2), 104.62 (C-3), 179.87 (C-4), 160.18 (C-5), 98.78 (C-6), 164.11 (C-7), 95.13 (C-8), 158.23 (C-9), 105.23(C-10), 120.43 (C-1′), 112.72 (C-2′), 146.01 (C-3′), 148.97 (C-4′), 116.11 (C-5′), 118.79 (C-6′), 98.73 (C-1′′), 72.41 (C-2′′), 75.78 (C-3′′), 67.45 (C-4′′), 76.20 (C-5′′), 61.35 (C-6′′).

Compound (3), *kaempferol 3-O-rutinoside* was obtained in the form of yellow crystals, which exhibited a melting point of 200 °C. R_*f*_ 0.89, The UV-λmax (MeOH) spectrum displayed absorption peaks at 257 nm and 370 nm, with an absorbance exceeding 350 nm, indicating the presence of a flavonol nucleus. When treated with MeOH + NaOMe, the compound exhibited a bathochromic shift with absorption peaks at 268 nm, 345 nm, and 415 nm, confirming the existence of polyhydroxyl groups. Furthermore, the addition of MeOH + AlCl_3_ resulted in a bathochromic shift with absorption peaks at 265 nm, 317 nm, and 420 nm, which could be attributed to the presence of a hydroxyl group at position C-5. The stability of the UV absorbance in band II was observed when MeOH + AlCl_3_/HCl was used, with absorption peaks at 264 nm, 315 nm (shoulder), and 360 nm, further confirming the presence of a hydroxyl group at position C-5. Treatment with MeOH + NaOAc led to a bathochromic shift in band II, with absorption peaks at 268 nm and 377 nm, indicating the presence of a free 7-OH group and a free 4’-OH group in band I. However, when MeOH + NaOAc/H_3_BO_3_ was employed, no bathochromic shift was observed in band I, suggesting the absence of *ortho*-dihydroxyl groups. In the ^1^H-NMR spectrum, signals were observed at 6.35 ppm (1H, s, H-6), 6.47 ppm (1H, s, H-8), 7.86 ppm (2H, d, H-3′, H-5′), and 8.02 ppm (2H, m, H-2′, H-6′), which were predicted to correspond to the kaempferol aglycon. Additionally, two anomeric proton signals were detected at 5.17 ppm (1H, d, H-1′′) and 4.59 ppm (1H, d, J = 1.2, H-1′′′), indicating the presence of two sugars. Several sugar protons were also observed in the range of 3.18–3.56 ppm (sugar protons, m). Furthermore, a signal at 0.98 ppm (3H, CH_3_ of rhamnosyl) was identified. The complete ^1^H- and ^13^C-NMR spectra supporting the structural elucidation of the isolated compounds are provided in Supplementary Figures (S5, S6, S7, S8, S9). Chemical structures of the major isolated flavonoids from *Viola odorata* flowers were presented in Supplementary Figure S10.

### In vitro anti-inflammatory evaluation

Cyclooxygenases, specifically COX-1 and COX-2, in conjunction with lipoxygenase (5-LOX), are acknowledged as key pro-inflammatory enzymes that are integral to the metabolism of arachidonic acid^51^. The anti-inflammatory properties of methanolic extract and isolated flavonoids from *V. odorata* flowers were evaluated by measuring their inhibitory effects on the enzymes COX-1, COX-2, and 5-LOX. This is illustrated in Table 3. The methanolic extract demonstrated remarkable inhibition of COX-1 and COX-2, with IC_50_ values of 1.623 ± 0.08 µg/ml and 1.506 ± 0.12 µg/ml, respectively, in comparison to the reference drug indomethacin, which exhibited IC_50_ values of 0.825 ± 0.14 µg/ml and 1.023 ± 0.09 µg/ml. Additionally, the extract effectively inhibited the 5-LOX enzyme, showing an IC_50_ of 1.024 ± 0.10 µg/ml, while Zileuton had an IC_50_ of 0.754 ± 0.12 µg/ml. Moreover, the isolated flavonoids exhibited significant anti-inflammatory effects, with kaempferol 3-*O*-rutinoside displaying the highest inhibitory potency against COX-1, COX-2, and 5-LOX enzymes, yielding IC_50_ values of 0.897 ± 0.10, 1.146 ± 0.10, and 0.793 ± 0.18 µg/ml, respectively. This was superior to the other two isolated flavonoids: luteolin 7-*O*-glucoside, which had IC_50_ values of 0.982 ± 0.09, 1.305 ± 0.14, and 0.915 ± 0.21 µg/ml, and 5,7-Dihydroxy-3,6-dimethoxyflavone, with IC_50_ values of 1.235 ± 0.12, 1.482 ± 0.02, and 0.987 ± 0.09 µg/ml, respectively.

**Table 3:** IC_50_ values of the methanolic extract and isolated compounds from *V. odorata* flowers against cyclooxygenase (COX-1 and COX-2) and 5-lipoxygenase (5-LOX) enzymes.

Tested group	IC_50_ values (ug/ml)			Selectivity Index (COX-1/COX-2)
	COX-1	COX-2	5-LOX	
Methanolic extract	1.623 ± 0.08^a^	1.506 ± 0.12^a^	1.024 ± 0.10^a^	1.08
5,7-Dihydroxy-3,6-dimethoxyflavone	1.235 ± 0.12^b^	1.482 ± 0.02^a^	0.987 ± 0.09^a^	0.83
Luteolin 7-*O*-glucoside	0.982 ± 0.09^c^	1.305 ± 0.14^b^	0.915 ± 0.21^ab^	0.75
Kaempferol 3-*O*-rutinoside	0.897 ± 0.10^c^	1.146 ± 0.10^c^	0.793 ± 0.18^b^	0.78
indomethacin	0.825 ± 0.14^c^	1.023 ± 0.09^c^		0.81
Zileuton			0.754 ± 0.12^b^	

The assessments were executed in sets of three (n = 3), and the findings were conveyed as mean ± standard error (SE). Superscript letters (ᵃ, ᵇ, ᶜ) indicate statistically significant differences within each column (*p* < 0.05, Tukey’s post hoc test). Groups sharing the same letter are not significantly different.

To assess the selectivity of the tested compounds toward COX isoforms, the selectivity index (SI) was calculated using the ratio of IC₅₀ values for COX-1 to COX-2. A higher SI indicates greater selectivity toward COX-1, while a lower SI suggests preferential inhibition of COX-2. These values suggest that the methanolic extract shows slight preference for COX-1, while the isolated flavonoids and indomethacin exhibit moderate selectivity toward COX-2, which may be beneficial in reducing gastrointestinal side effects associated with COX-1 inhibition.

### Cytotoxic evaluation

In this investigation, the cytotoxic effects of the methanolic extract and isolated flavonoids were assessed against hepatocellular carcinoma (HepG2), human colonic epithelial cells (Caco-2), and human colorectal carcinoma cells (HTC-116) through the application of the MTT assay. Detailed results are presented in Table 4. The findings revealed that the methanolic extract of *V. odorata* flowers demonstrated a considerable cytotoxic effect in a dose-dependent manner, with IC_50_ values of 48.11, 42.42, and 38.65 µg/mL against the proliferation of HepG2, Caco-2, and HTC-116 cells, respectively, when compared to doxorubicin, which had IC_50_ values of 23.55, 27.13, and 25.73 µg/mL. Additionally, all isolated flavonoids exhibited significant cytotoxicity across liver, colon, and colorectal cell lines, with kaempferol 3-*O*-rutinoside displaying the most potent inhibitory effects (IC_50_ values of 25.78, 24.59, and 25.18 µg/mL), surpassing those of 5,7-dihydroxy-3,6-dimethoxyflavone (IC_50_ values of 32.02, 32.81, and 31.86 µg/mL) and luteolin 7-*O*-glucoside (IC_50_ values of 28.23, 29.42, and 30.17 µg/mL). The cytotoxic potency is likely due to the presence of multiple hydroxyl groups and sugar moieties, enhancing cell permeability and interaction with cellular targets. These results underscore the chemopreventive potential of *V. odorata*-derived flavonoids.

**Table 4:** Percentage of cell viability and cytotoxicity of the methanolic extract and the isolated favonoids against hepatocellular carcinoma (hepG2), human colonic epithelial cells (Caco-2) and human colorectal carcinoma cells (HTC-116) compared to doxorubicin as standard.

	Tested cell line	HepG2					
	Concentration (ug/ml)	100	50	25	12.5	6.25	IC_50_
Methanolic extract	% Cell viability	24.76	46.24	61.01	65.75	76.55	48.11^a^
	% Cytotoxicity	75.24	53.76	38.99	34.25	23.45	
5,7-Dihydroxy-3,6-dimethoxyflavone	% Cell viability	16.49	30.12	44.07	64.86	75.11	32.02^b^
	% Cytotoxicity	83.51	69.88	55.93	35.41	24.89	
Luteolin 7-*O*-glucoside	% Cell viability	14.11	28.52	42.58	63.99	70.35	28.23^c^
	% Cytotoxicity	85.89	71.48	57.42	36.01	29.65	
Kaempferol 3-*O*-rutinoside	% Cell viability	8.57	23.88	41.08	64.22	70.44	25.78^c^
	% Cytotoxicity	91.43	76.12	58.92	35.78	29.56	
Doxorubicin	% Cell viability	7.95	24.01	39.86	62.31	68.24	23.55^c^
	% Cytotoxicity	92.05	75.99	60.14	37.69	31.76	

	Tested cell line	Caco-2					
	Concentration (ug/ml)	100	50	25	12.5	6.25	IC_50_
Methanolic extract	% Cell viability	21.65	43.73	59.74	63.83	70.44	42.42^a^
	% Cytotoxicity	78.35	56.27	40.26	36.17	29.56	
5,7-Dihydroxy-3,6-dimethoxyflavone	% Cell viability	13.26	33.48	51.09	62.16	72.17	32.81^b^
	% Cytotoxicity	86.74	66.52	48.91	37.84	27.83	
Luteolin 7-*O*-glucoside	% Cell viability	10.63	31.01	49.22	60.35	70.32	29.42^c^
	% Cytotoxicity	89.37	68.99	50.78	39.65	29.68	
Kaempferol 3-*O*-rutinoside	% Cell viability	6.33	27.35	44.63	56.22	70.58	24.59^c^
	% Cytotoxicity	93.67	72.65	55.37	43.78	29.42	
Doxorubicin	% Cell viability	7.87	28.76	46.11	58.44	71.87	27.13^c^
	% Cytotoxicity	92.13	71.24	53.89	41.56	28.13	

	Tested cell line	HTC-116					
	Concentration (ug/ml)	100	50	25	12.5	6.25	IC_50_
Methanolic extract	% Cell viability	24.02	40.33	56.33	61.54	67.55	38.65^a^
	% Cytotoxicity	75.98	59.67	43.67	38.46	32.45	
5,7-Dihydroxy-3,6-dimethoxyflavone	% Cell viability	14.08	35.12	50.13	60.83	70.17	31.86^b^
	% Cytotoxicity	85.92	64.88	49.87	39.17	29.83	
Luteolin 7-*O*-glucoside	% Cell viability	13.01	34.03	49.17	59.38	69.86	30.17^c^
	% Cytotoxicity	86.99	65.97	50.83	40.62	30.14	
Kaempferol 3-*O*-rutinoside	% Cell viability	10.55	31.04	46.25	55.21	68.33	25.18^c^
	% Cytotoxicity	89.45	68.96	53.75	44.79	31.67	
Doxorubicin	% Cell viability	8.77	30.02	46.19	56.90	68.98	25.73^c^
	% Cytotoxicity	91.23	69.98	53.81	43.10	31.02	

The assessments were executed in sets of three (n = 3), and the findings were conveyed as mean ± standard error (SE). Superscript letters (ᵃ, ᵇ, ᶜ) indicate statistically significant differences within each column (*p* < 0.05, Tukey’s post hoc test). Groups sharing the same letter are not significantly different.

### Molecular dynamic and system stability

**Fig. 1:**
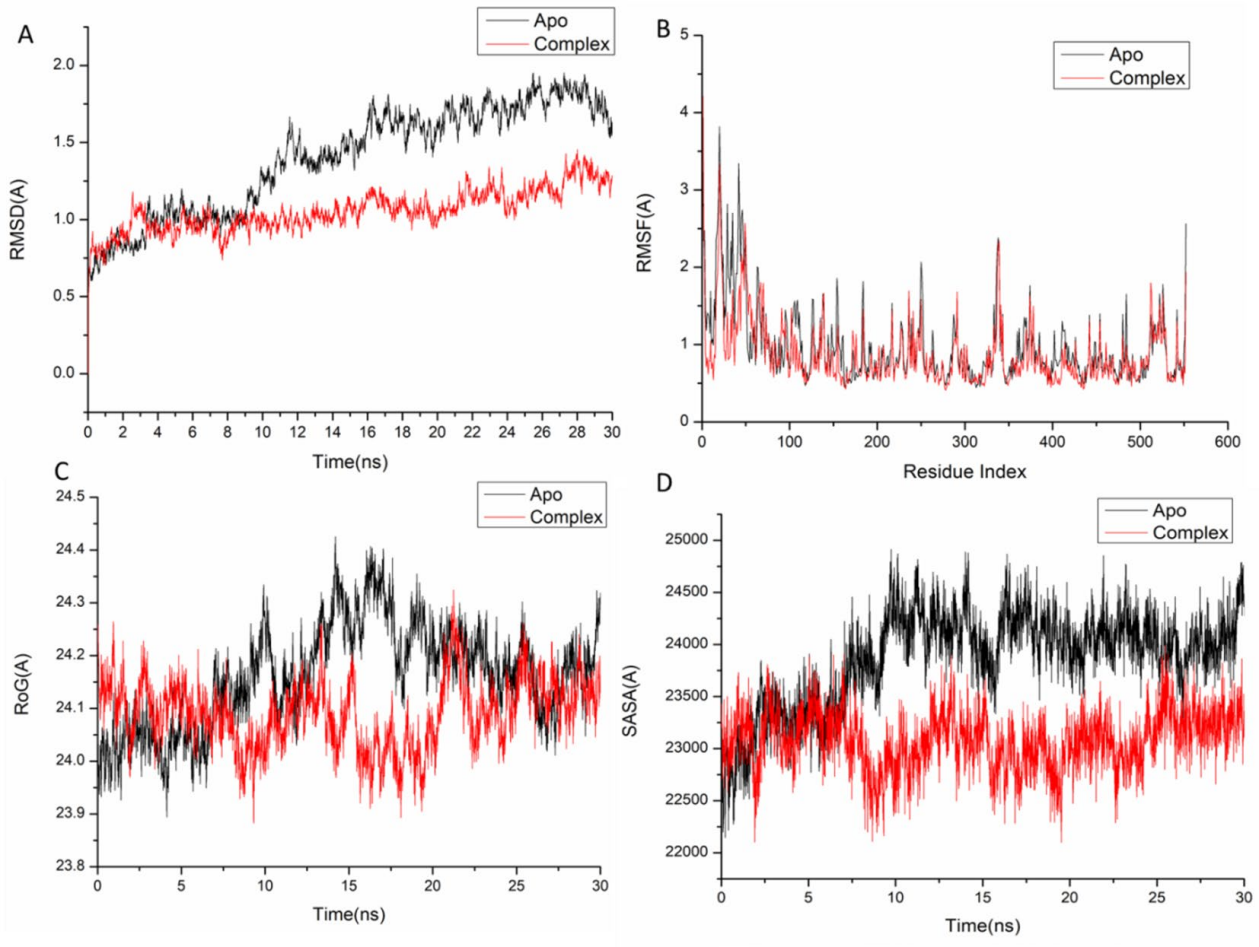
(**A**) RMSD of Cα atoms of the protein backbone atoms. [B] RMSF of each residue of the protein backbone Cα atoms of protein residues [C] ROG of Cα atoms of protein residues; [D] solvent accessible surface area (SASA) of the C α of the backbone atoms relative (black) to the starting minimized over 50 ns for the catalytic binding site with Kaempferol 3-*O*-rutinoside – COX-2 complex system (red).

**Fig. 2:**
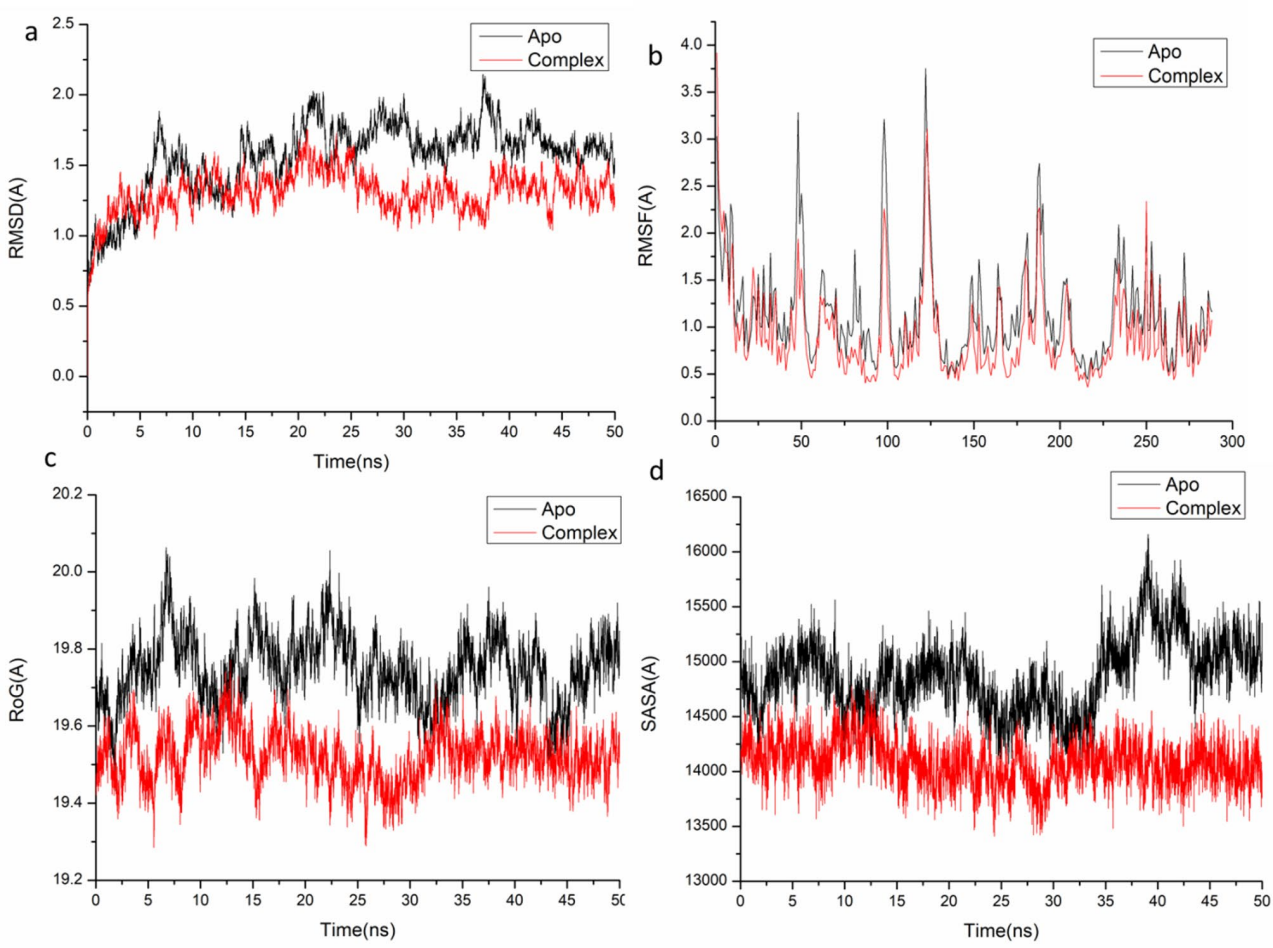
(**A**) RMSD of Cα atoms of the protein backbone atoms. (**B**) RMSF of each residue of the protein backbone Cα atoms of protein residues (**c**) ROG of Cα atoms of protein residues; (**d**) solvent accessible surface area (SASA) of the C α of the backbone atoms relative (black) to the starting minimized over 50 ns for the catalytic binding site with Kaempferol 3-*O*-rutinoside – VEGFR complex system (red).

The performance of isolated flavonoids in relation to their binding at the active site of a protein was investigated through a molecular dynamics simulation, which also examined their interactions and stability^52,53^. Validating the stability of the system is vital for detecting any disrupted motions and mitigating potential artifacts that may occur throughout the simulation. The present study investigated the Root-Mean-Square Deviation (RMSD) to evaluate the stability of the systems during the simulation process. The average RMSD values obtained for the apo-protein and Kaempferol 3-*O*-rutinoside – COX-2 systems were found to be 1.40 ± 0.34 Å and 1.05 ± 0.13 Å, respectively. Additionally, the apo-protein and Kaempferol 3-*O*-rutinoside – VEGFR-1 systems demonstrated average RMSD values of 1.57 ± 0.23 Å and 1.32 ± 0.15 Å, as depicted in Figs. 1A. The findings indicated that the protein complex system associated with Kaempferol 3-*O*-rutinoside exhibited a comparatively more stable conformation than the other systems examined. In molecular dynamics (MD) simulations, it is essential to evaluate the structural flexibility of proteins in response to ligand binding, as this analysis provides insights into the behavior of specific residues and their interactions with the ligand^54^. The fluctuations of protein residues were assessed using the Root-Mean-Square Fluctuation (RMSF) method to determine the impact of inhibitor binding on the corresponding targets throughout the simulations. The average RMSF values calculated for all frames of the apo-protein and the Kaempferol 3-*O*-rutinoside – COX-2 systems were found to be 0.98 ± 0.50 Å and 0.86 ± 0.42 Å, respectively. Refer to Fig. 1B for visual representation. The root mean square deviations for the apo-protein and Kaempferol 3-*O*-rutinoside – VEGFR-1 systems were determined to be 1.15 ± 0.53 Å and 0.92 ± 0.48 Å, respectively. As depicted in Fig. 2, these findings suggest that the Kaempferol 3-*O*-rutinoside complex exhibits less residue fluctuation compared to the other systems analyzed.

ROG aimed to assess the compactness and stability of the system in response to ligand binding during molecular dynamics simulations^55,56^. The average radius of gyration (Rg) for the apo-protein and the Kaempferol 3-*O*-rutinoside – COX-2 systems were recorded at 24.16 ± 0.09 Å and 24.09 ± 0.06 Å, respectively (Fig. 1C). On the other hand, the Rg values for the apo-protein and the Kaempferol 3-*O*-rutinoside – VEGFR-1 systems were 19.74 ± 0.08 Å and 19.51 ± 0.06 Å, respectively. The data suggest that the complex formed with Kaempferol 3-*O*-rutinoside exhibits a notably rigid structure in relation to the catalytic binding site of the target receptor.

The assessment of the protein hydrophobic core’s compactness was conducted through the calculation of the solvent accessible surface area (SASA). This involved quantifying the surface area of the protein that is exposed to the solvent, a critical factor for the stability of biomolecules^57^. The average SASA values obtained for the apo-protein and the Kaempferol 3-*O*-rutinoside – COX-2 systems were 23,873.34 Å and 23,077.99 Å, respectively. (Fig. 1D). In contrast, the SASA values for the apo-protein and the Kaempferol 3-*O*-rutinoside – VEGFR-1 systems were 14,926.07 Å and 14,051.54 Å, respectively. The SASA results, when analyzed alongside the findings from the RMSD, RMSF, and ROG calculations, provided evidence that the Kaempferol 3-*O*-rutinoside complex remains stable within the catalytic binding site of the target receptor.

### Binding interaction mechanism based on binding free energy calculation

The molecular mechanics energy technique (MM/GBSA) is a widely utilized approach for assessing the free binding energies of small molecules interacting with biological macromolecules. This method integrates generalized Born and surface area continuum solvation, potentially offering greater reliability than traditional docking scores^58^. In this study, the MM-GBSA module within AMBER18 was employed to compute the binding free energies by extracting snapshots from the system trajectories. As presented in Table 5, all calculated energy components, with the exception of ΔGsolv, exhibited significantly negative values, suggesting favorable interactions.

**Table 5:** The calculated energy binding for the Kaempferol 3-*O*-rutinoside against the catalytic binding site of target receptor.

Complex	ΔE_vdW_	ΔE_elec_	ΔG_gas_	ΔG_gas_	ΔG_bind_
Energy components (kcal/mol)					
COX-2					
Kaempferol 3-*O*-rutinoside –COX2	− 68.72 ± 0.44	− 48.18 ± 0.81	− 116.91 ± 0.91	− 116.91 ± 0.91	− 60.78 ± 0.56
VEGFR					
Complex	ΔE_vdW_	ΔE_elec_	ΔG_gas_	ΔG_gas_	ΔG_bind_
Kaempferol 3-*O*-rutinoside –VEGFR	− 69.75 ± 0.37	− 54.81 ± 0.23	− 114.93 ± 0.91	− 114.93 ± 0.91	− 49.76 ± 0.46

∆EvdW, van der Waals energy; ∆Eele, electrostatic energy; ∆Gsolv, solvation free energy; ∆Gbind, calculated total binding free energy.

The binding interactions between Kaempferol 3-O-rutinoside and the residues of the target protein receptor are primarily influenced by the more favorable Van der Waals energy component. This conclusion is supported by a comprehensive analysis of the individual energy contributions, which ultimately informs the observed binding free energies.

### Identification of the critical residues responsible for ligands binding

**Fig. 3:**
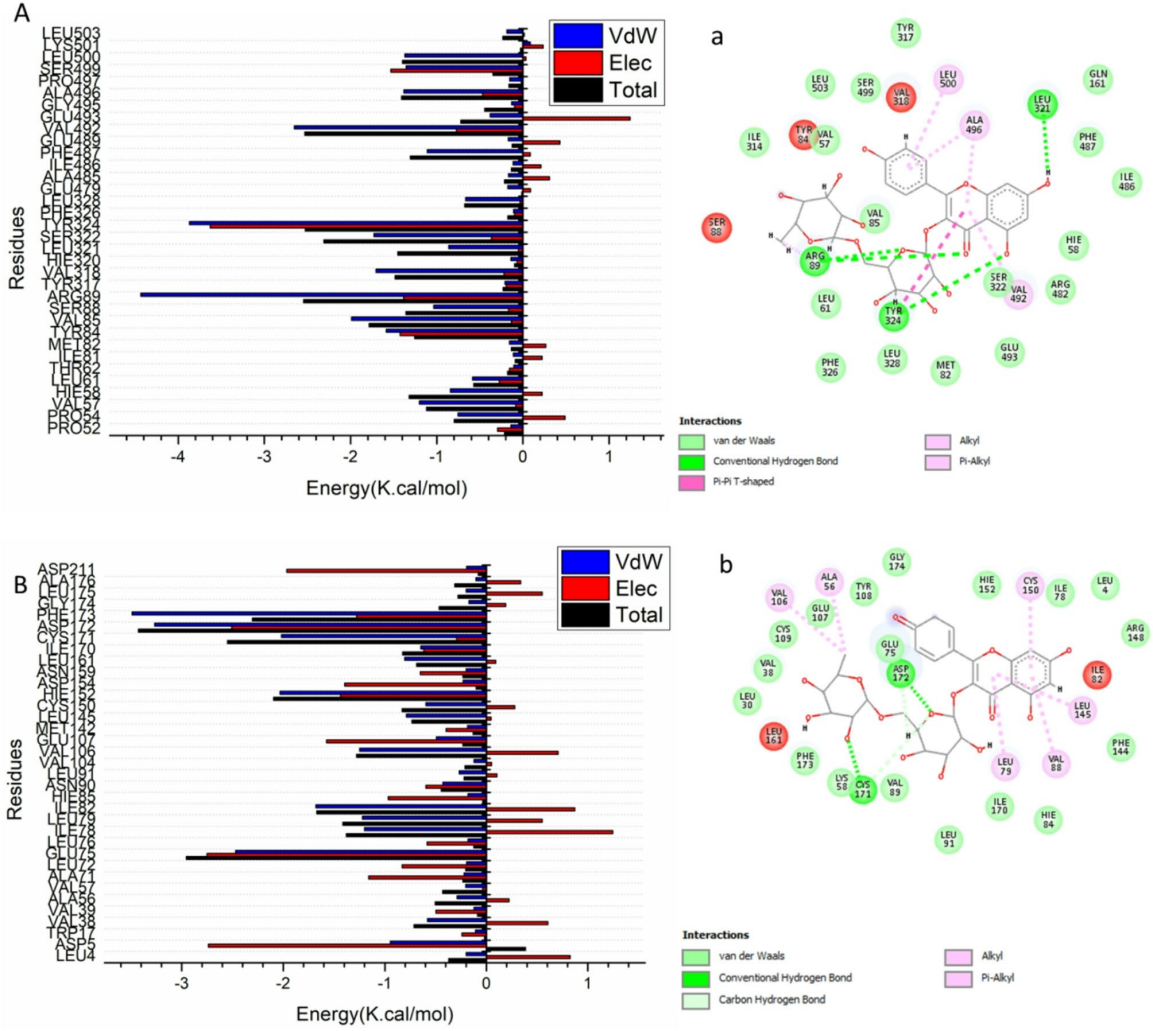
Per-residue decomposition plots showing the energy contributions to the binding and stabilization of Kaempferol 3-*O*-rutinoside into catalytic binding site of COX-2 receptor (**A**), VEGR-1 receptor (**B**).Corresponding inter-molecular interactions are shown (**a**), (**b**).

The overall energy associated with the binding of the compound to the enzymes was analyzed by breaking it down into the contributions of individual site residues. This approach aimed to enhance our understanding of the critical residues that play a role in inhibiting the catalytic binding site of the receptor. As illustrated in Fig. 3, the primary favorable contributions of Kaempferol 3-*O*-rutinoside to the catalytic binding site of the COX-2 receptor are notably derived from the residues Pro 54 (− 0.753 kcal/mol) and Val 57 (− 1.203 kcal/mol), Hie 58 (− 0.843 kcal/mol), Leu 61 (− 0.586 kcal/mol), Thr 62 (− 0.106 kcal/mol), Met 82 (− 0.155 kcal/mol), Tyr 84 (− 1.585 kcal/mol), Val85 (− 1.991 kcal/mol), Ser 88 (− 1.035 kcal/mol), Arg 89 (− 4.434 kcal/mol), Tyr 317 (− 0.209 kcal/mol), Val 318 (− 1.706 kcal/mol), Leu 321 (− 0.862 kcal/mol), Ser 322 (− 1.731 kcal/mol), Tyr 324 (− 3.869 kcal/mol), Leu 328 (− 0.664 kcal/mol), Glu 479 (− 0.175 kcal/mol), Phe 487 (− 1.111 kcal/mol), Val 492 (− 2.652 kcal/mol), Glu 493 (− 0.381 kcal/mol), Ala 496 (− 1.382 kcal/mol), Pro 497 (− 0.154 kcal/mol), Ser 499 (− 1.356 kcal/mol), Leu 500 (− 1.371 kcal/mol), and Leu 503 (− 0.186 kcal/mol).

In another perspective, as shown in Fig. 2, the significant favorable effect of Kaempferol 3-*O*-rutinoside on the catalytic binding site of the VEGFR receptor is predominantly associated with the residues Leu 4 (− 0.199 kcal/mol) and Asp 5 (− 0.946 kcal/mol), Val 38 (− 0.579 kcal/mol), Ala 56 (− 0.289 kcal/mol), Val 57 (− 0.203 kcal/mol), Ala 71 (− 0.22 kcal/mol), Leu 72 (− 0.195 kcal/mol), Glu 75 (− 2.469 kcal/mol), Ile 78 (− 1.201 kcal/mol), Leu 79 (− 1.221 kcal/mol), Ile 82 (− 1.679 kcal/mol), Asn 90 (− 0.429 kcal/mol), Leu 91 (− 0.267 kcal/mol), Val 106 (− 1.248 kcal/mol), Glu 107 (− 0.493 kcal/mol), Leu 145 (− 0.789 kcal/mol), Cys 150 (− 0.596 kcal/mol), Hie 152 (− 2.032 kcal/mol), Asp 154 (− 0.228 kcal/mol), Asn 159 (− 0.199 kcal/mol), Leu 161 (− 0.805 kcal/mol), Ile 170 (− 0.647 kcal/mol), Cys 171 (− 2.018 kcal/mol), Asp 172 (− 3.267 kcal/mol), Phe 173 (− 3.49 kcal/mol), Leu 175 (− 0.199 kcal/mol),and Asp 211 (− 0.194 kcal/mol).

#### Ligand–residue interaction network profiles

**Fig. 4:**
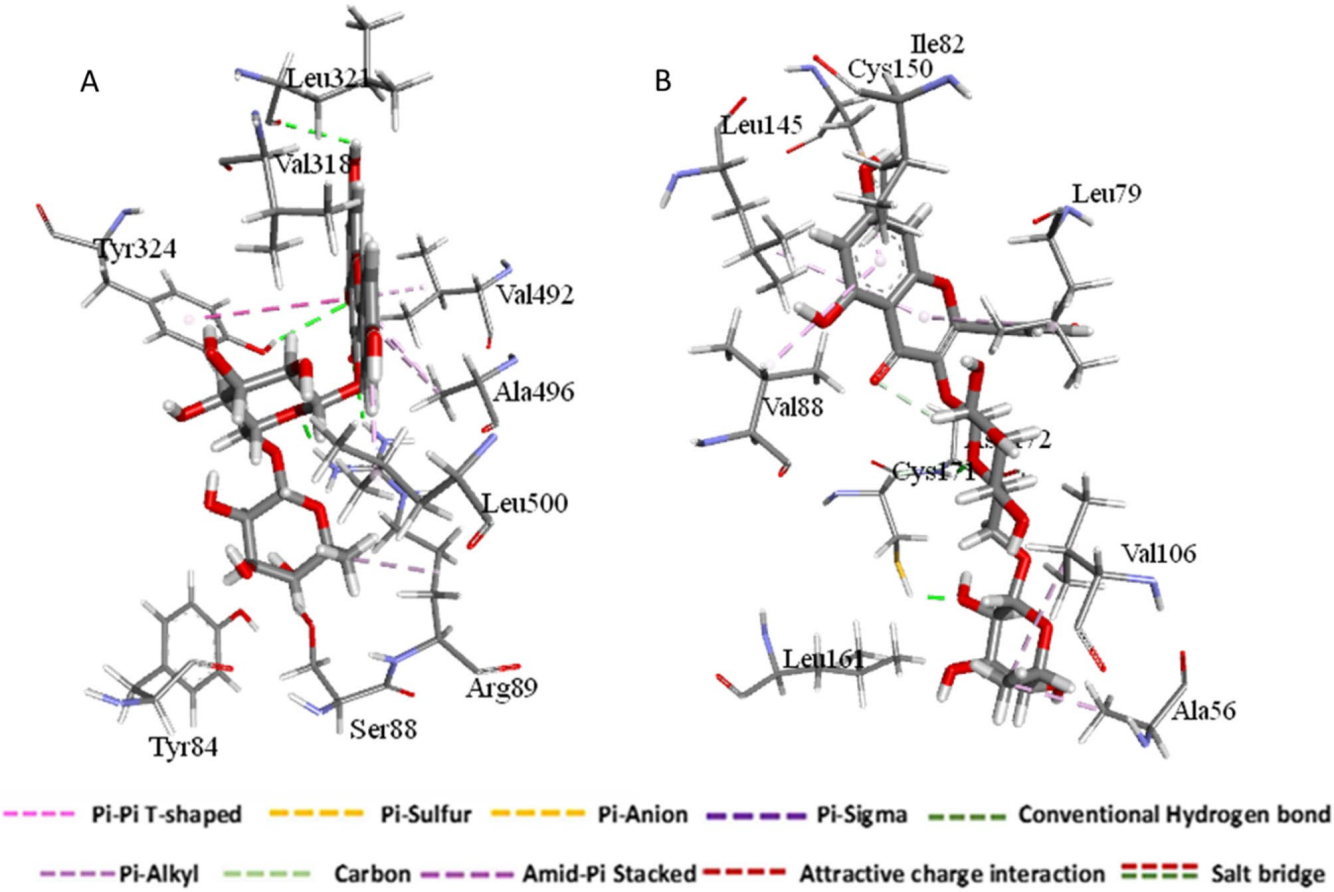
The interaction residue of Kaempferol 3-*O*-rutinoside into the catalytic binding site of COX-2 receptor (**A**), and VEGFR-1 (**B**) using BIOVIA Discovery Studio Visualizer v25.1.0.24284 (Dassault Systèmes, San Diego, CA, USA; https://discover.3ds.com/discovery-studio-visualizer-download).

A key objective in the field of drug design is to modify the structure of therapeutic compounds to enhance bioavailability, minimize toxicity, and optimize pharmacokinetic properties^59^. Kaempferol 3-*O*-rutinoside has demonstrated a stable hydrogen bonding interaction with Leu 321 and Arg 89. Furthermore, an api-alkyl interaction has been established between Kaempferol 3-*O*-rutinoside and Leu 500, as well as Ala 496. The pharmacophoric hotspot residue Tyr 324 has participated in both Pi-pi T-shaped and hydrogen bonding interactions with Kaempferol 3-*O*-rutinoside. Furthermore, Kaempferol 3-*O*-rutinoside has exhibited a stable hydrogen bonding interaction with Cys 171 and Asp 172. Additionally, an api-alkyl interaction was noted between Kaempferol 3-*O*-rutinoside and the residues Cys 150, Val 88, Leu 79, Ala 56, and Val 106 (Fig. 4).

#### Principal component analysis (PCA)

**Fig. 5:**
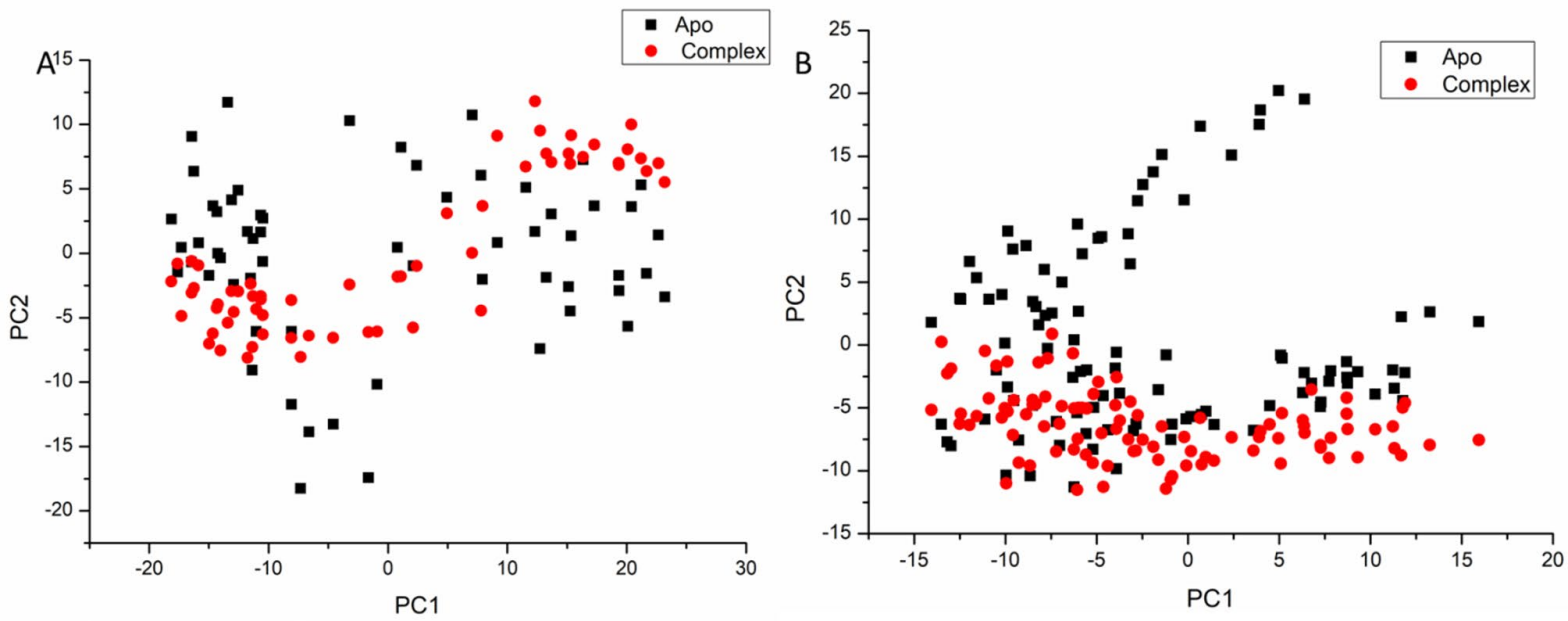
PCA projection of Cα atoms motion constructed by plotting the first two principal components (PC1 andPC2) in conformational space, apo (black ), Kaempferol 3-*O*-rutinoside (red) for COX-2 receptor (**A**), and VEGFR-1 (**B**) respectively.

The PCA plot presented in Fig. 5 illustrates the presence of distinct and intricate conformational waves across the two primary components within a notable subspace. The apo-protein and the Kaempferol 3-*O*-rutinoside complex systems displayed a marked differentiation in their dynamic behavior. Consequently, the eigenvector analyses derived from the 50 ns molecular dynamics trajectories for these three systems reveal significant variations, underscoring the differences in protein dynamics. The interaction of the ligand with the active sites of the Kaempferol 3-*O*-rutinoside complex system likely triggers conformational changes, which are subsequently represented by the principal components as a wave-like motion. This observation is linked to the apo system displaying greater atomic variability than the complex system. The Kaempferol 3-*O*-rutinoside-complex system is distinguished by its higher density of molecular packing. Thus, when Kaempferol 3-*O*-rutinoside interacts with the protein, there is a notable reduction in conformational flexibility, which enhances the ligand’s binding to the active site.

#### Dynamics cross-correlation matrices (DCCM) analysis

**Fig. 6:**
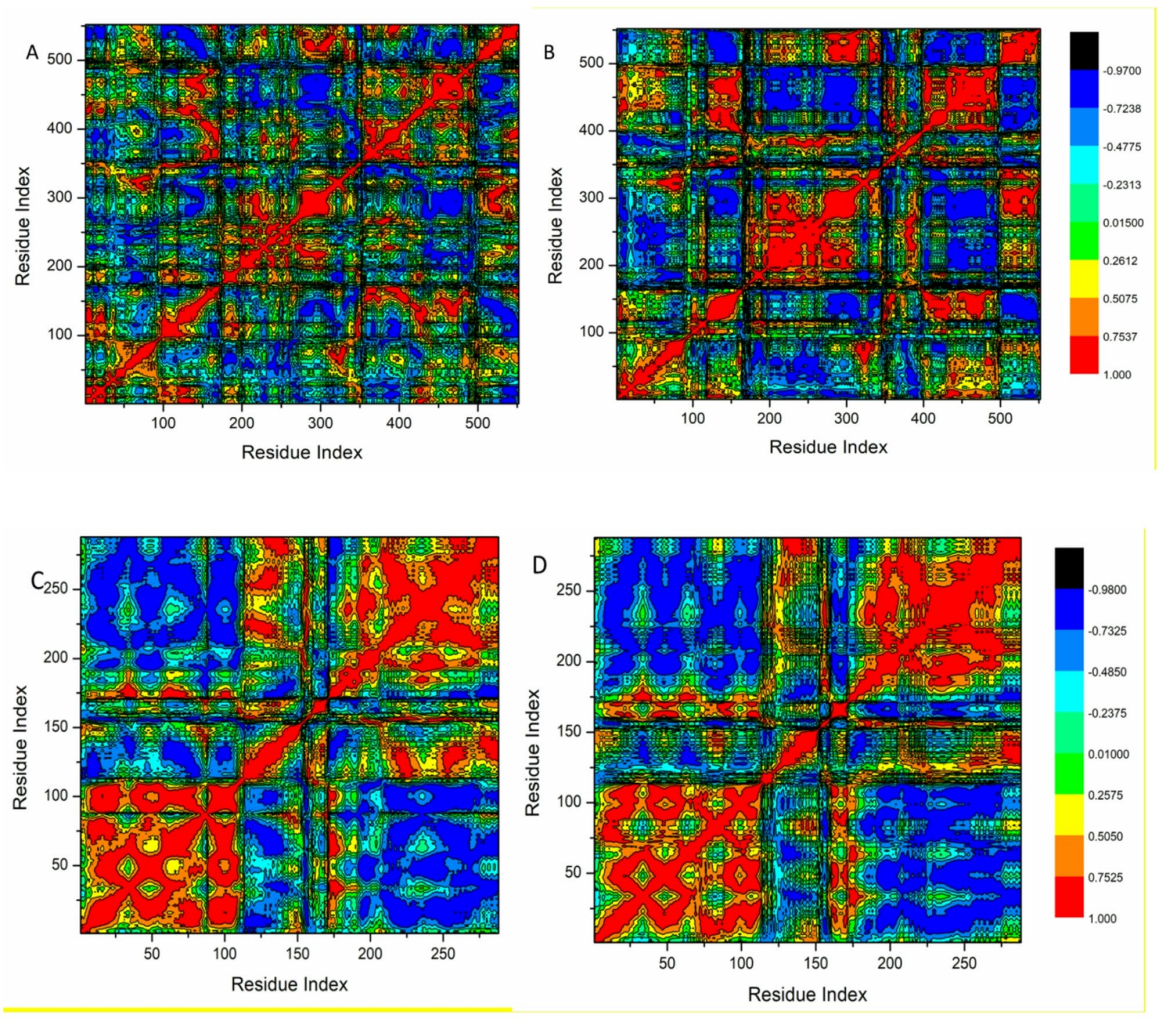
Dynamic cross-correlation matrix analyses for the Apo- COX-2 (**A**), and Kaempferol 3-*O*-rutinoside—COX-2 receptor complex systems (**B**), and for the Apo- VEGFR-1 (**C**), and Kaempferol 3-*O*-rutinoside—VEGFR-1 receptor complex systems (**D**), respectively.

The analysis of DCCM was conducted on the Cα positions across the simulations to assess the conformational alterations of Cox-2 and the resulting interactions with ligands, in addition to examining the dynamics and associated motions present (Fig. 6). The areas marked in yellow–red represent residues exhibiting strong positive correlations in their movements, whereas the regions depicted in blue-black signify residues that demonstrate strong negative correlations in their motions. The systems examined in this research demonstrated a tendency for residues to exhibit correlated motions rather than anti-correlated ones. The interaction of Kaempferol 3-*O*-rutinoside with the COX-2 and VEGFR-1 proteins affects the structural dynamics of these proteins, resulting in conformational alterations that are evident in the changes of their associated movements, as indicated by the DCCM analysis.

The data presented in Fig. 5A and B clearly indicate that Kaempferol 3-*O*-rutinoside engages with COX-2 proteins at particular sites, with significant correlations noted in the residue ranges of 200–300 and 400–500. Additionally, Fig. 5C and D demonstrate that Kaempferol 3-*O*-rutinoside also associates with VEGFR-1 proteins in analogous regions, revealing important correlations within the 200–250 residue range of the VEGFR-1 protein.

#### Free energy landscape

**Fig. 7:**
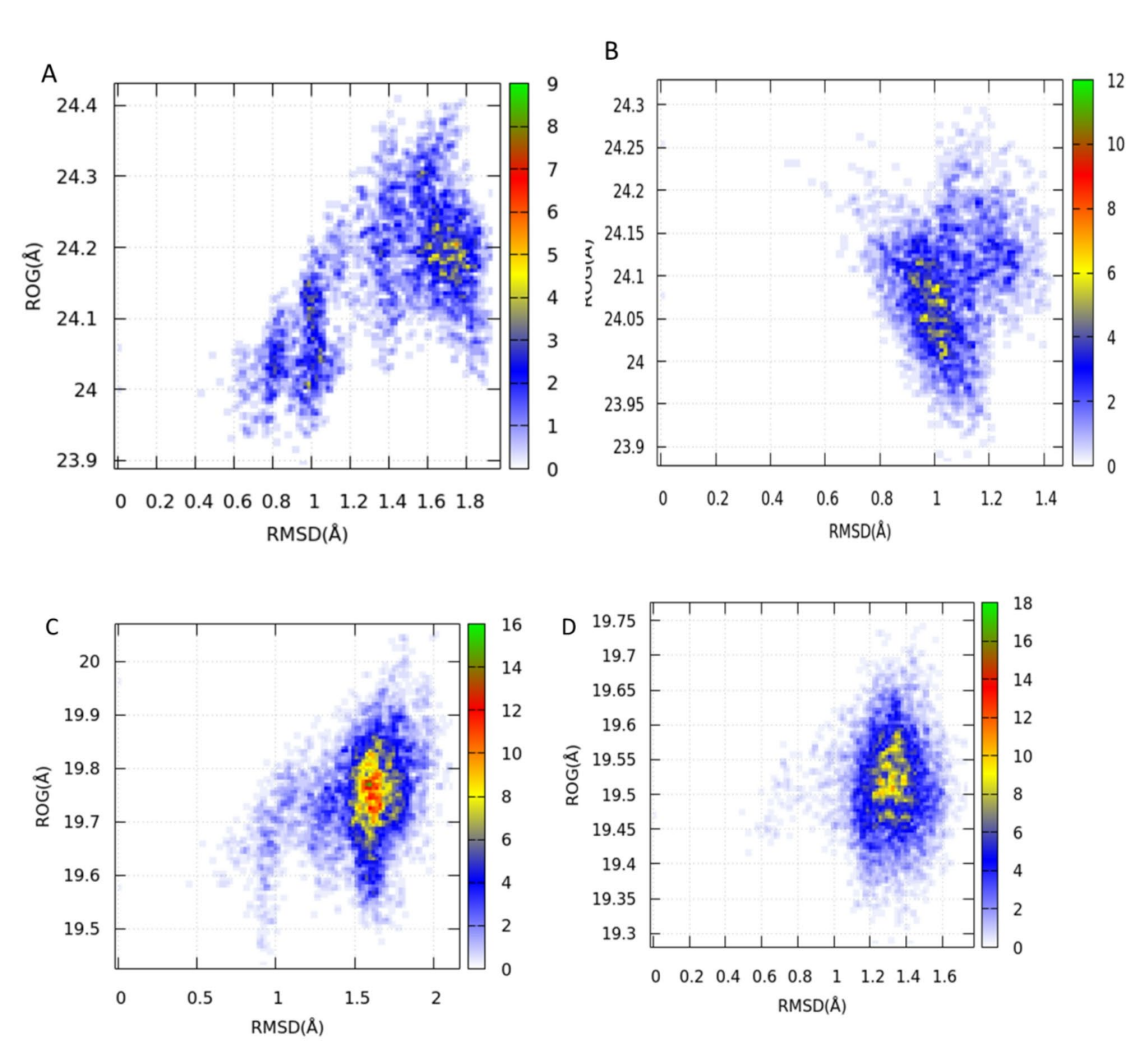
Conformational free energy landscape for COX2-Apo (**A**), COX2- Kaempferol 3-*O*-rutinoside (**B**), VEGFR-1 -Apo (**C**), and VEFGR-1- Kaempferol 3-*O*-rutinoside systems.

Two-dimensional free energy contour maps have been developed to depict the correlation between RMSD and RoG for Kaempferol 3-*O*-rutinoside in its interactions with the COX-2 and VEGFR-1 systems. These maps feature representative structures that correspond to each local free energy minimum, thereby enabling an examination of the impact of this compound on the misfolding of COX-2 and VEGFR-1 proteins (Fig. 6). The complexes formed by Kaempferol 3-*O*-rutinoside with COX-2 and VEGFR-1 exhibited a considerable conformational space when compared to the Apo system (Fig. 7). Overall, these results suggest that Kaempferol 3-*O*-rutinoside significantly influences the free energy landscape of both COX-2 and VEGFR-1 proteins.

## Discussion

Currently, scientists worldwide are growing more fascinated by the potential of plant-derived medications in the prevention and treatment of diverse ailments. In the present study, the comprehensive phytochemical composition of *V. odorata* flowers were investigated, with a specific focus on the flavonoid and anthocyanin constitution.

The total phenolic, flavonoid and anthocyanin contents in the methanolic extract of *V. odorata* flowers were assessed, and the outcomes align with the findings reported by Habibi et al.^60^ and Zawiślak et al.^61^. These results emphasize the presence of abundant polyphenols and anthocyanins in *V. odorata* flowers. Consequently, the identification of phenolic acids and flavonoid components in the methanol extract was conducted utilizing HPLC and LC–ESI–MS negative and positive ionization techniques. The finding revealed the presence of a diverse array of active constituents, consisting of 8 phenolic acids and their derivatives, 3 flavonols, 4 flavones, and 14 flavonoid glycosides. Additionally, five anthocyanins derived from aglycones cyanidin, delphinidin, and petunidin were also identified. Notably, the high anthocyanin content suggests potential antioxidant benefits, possibly contributing to the various pharmacological activities. Ccompounds such as quercetin, kaempferol, and luteolin derivatives, all known for strong bioactivities, were detected, highlighting the pharmacological value of *V. odorata* flowers.

In the negative ionization mode, a tentative identification of five phenolic acids and three derivatives was achieved in the flowers of *V. odorata*. These compounds demonstrated a consistent fragmentation pathway, which involved the elimination of a CO_2_ group and water, as detailed by Ristivojević et al.^62^. Of particular interest, ferulic acid was noted to also undergo the loss of a methyl group, leading to the formation of a fragment ion at *m/z* 178, as reported by Gardana et al.^63^. A comprehensive examination of the mass fragmentation patterns of flavonoids reveals that these compounds adhere to the RDA fragmentation pathway, predominantly yielding [1,3A]- and [1,2A]- fragments^64^, and loss of H_2_O and CO molecules^65^. This fragmentation behavior was observed in quercetin and myricetin. Isorhamnetin exhibited a comparable fragmentation pattern, with loss of a methyl group^66^**.** On the other hand, in the positive ionization mode, the methoxy flavones, specifically 6-Hydroxy 3’,4’,5,7,8-pentamethoxy flavone, 4’,5,6,7-tetramethoxy flavone, and 5,7,4’-trimethoxyflavone, underwent fragmentation characterized by the loss of methyl groups, leading to the formation of ions at *m/z* 374, 328, and 298, respectively^65^. Additionally, the flavone luteolin produced a fragment at *m/z* 269 as a result of water loss, a finding corroborated by previous studies^66^.

The identification of flavonoid glycosides was succeeded by cleaving the glycosidic *O*-linkages, which resulted in the release of monosaccharide residues along with H-rearrangement. In the case of hesperidin, luteolin 7-*O*-rutinoside, apigenin 7-*O*-rutinoside, rutin, kaempferol 3-*O*-rutinoside, and isorhamnetin 3-*O*-rutinoside, the removal of rutinoside produced a fragment peak that corresponded to the aglycone^66^. On the other hand, apigenin 7-*O*-glucoside, kaempferol 3-*O*-glucoside, quercetin 3-*O*-glucoside, luteolin 8-*O*-glucoside, and myricetin 3-*O*-glucoside underwent mass fragmentation by removing glucoside, while kaempferol 3-*O*-arabinoside and quercetin 3-*O*-arabinoside eliminated arabinose^67^. The structural identification of the flavonoids found in Viola species was consistent with previous studies conducted by Karioti et al.^50^ and Papp et al.^68^. It is worth noting that isorhamnetin, luteolin^69^, quercetin, rutin, and luteolin 7-*O*-glucoside^68^ were the predominant flavonoids in *V. odorata* flowers, as well as quercetin 3-*O*-glucoside and myricetin 3-*O*-glucoside^70^.

The violet color of *V. odorata* flowers can be attributed to the presence of various anthocyanins, which are glycosides of anthocyanidins. Through positive mode analysis, five distinct anthocyanins were identified, originating from the aglycones cyanidin, delphinidin, and petunidin. This identification was based on the detection of specific fragment signals at *m/z* 287, 303, and 317, respectively, during mass fragmentation analysis^47^. Each of these anthocyanins exhibited a typical anthocyanin fragmentation pattern. The identification process is further corroborated by references in the scientific literature^47,50^.

Additionally, three flavonoids were successfully isolated, and their structures were elucidated through a range of spectroscopic methods. The first compound, identified as 5,7-dihydroxy-3,6-dimethoxyflavone, had been previously isolated by Karim et al.^71^ from the whole plant of *V. odorata* in Pakistan. The second compound was identified as luteolin 7-*O*-glucoside by comparing the acquired data with existing literature^72^ and considering the melting point^73^. The third compound, kaempferol 3-*O*-rutinoside, was identified by correlating the spectroscopic findings with the data available in the scientific literature for various flavonol glycosides^74^.

Subsequently, the anti-inflammatory properties of the methanolic extract and isolated flavonoids from the flowers of *V. odorata* were evaluated. The methanolic extract showed notable inhibition of both COX-1 and COX-2 enzymes when compared to the reference drug indomethacin. The selectivity index showed moderate COX-2 preference, suggesting reduced gastrointestinal toxicity relative to non-selective NSAIDs. These findings suggest that the polyphenolic profile directly contributes to the anti-inflammatory activity of *V. odorata*. Furthermore, the extract effectively inhibited the 5-LOX enzyme in comparison to Zileuton. Additionally, the isolated flavonoids demonstrated significant anti-inflammatory effects, with kaempferol 3-*O*-rutinoside exhibiting the highest inhibitory activity against COX-1, COX-2, and 5-LOX enzymes, surpassing the effects of the other two isolated flavonoids: luteolin 7-*O*-glucoside and 5,7-dihydroxy-3,6-dimethoxyflavone.

Inflammation is believed to play a crucial role in the development of cancer^75^. Inflammatory cells, along with chemokines and cytokines, have been identified in all tumor microenvironments examined in both experimental animal models and human studies from the earliest stages of tumorigenesis. Research has demonstrated both in vitro and in vivo anti-inflammatory properties of flavonoids, particularly kaempferol, its glycosides, and extracts containing kaempferol^76^. The anti-inflammatory effects of kaempferol may operate through various mechanisms. It has been shown to inhibit the phosphorylation of PI3K and AKT induced by LPS and ATP in cardiac fibroblasts, thus safeguarding these cells from inflammatory damage^77^. Furthermore, kaempferol significantly reduces the production of nitric oxide and tumor necrosis factor-alpha (TNF-α) in RAW 264.7 cells that have been stimulated by LPS^78^.

The anti-cancer properties of the methanolic extract of *V. odorata* flowers, along with the isolated flavonoids, were assessed using various cell line models. Our results indicated that the methanolic extract exhibited a dose-dependent cytotoxicity, with IC_50_ values of 48.11, 42.42, and 38.65 µg/mL against the proliferation of hepatocellular carcinoma (HepG2), human colonic epithelial cells (Caco-2), and human colorectal carcinoma cells (HTC-116), respectively. These findings align with earlier studies that have highlighted the cytotoxic effects of the aerial parts of *Viola odorata*^12^ and other Viola species, such as *Viola tricolor*^79^, which have demonstrated inhibitory effects on the growth of MCF-7 and Neuro2a cell lines. Furthermore, the isolated flavonoids exhibited notable cytotoxic effects in liver, colon, and colorectal cell lines, with kaempferol 3-*O*-rutinoside showing the most potent inhibitory effects compared to 5,7-dihydroxy-3,6-dimethoxyflavone and luteolin 7-*O*-glucoside.

The presence of a diverse array of flavonoids and anthocyanins in *V. odorata* flowers has been attributed to their remarkable anticancer properties. Flavonoids have been reported to possess a wide range of biological benefits, including anti-infammatory, anti-microbial, anti-arthritic, anti-diabetic and cardioprotective effects^25,80^. Furthermore, they have been associated with protection against diseases linked to oxidative stress, such as Alzheimer’s, Parkinson’s, atherosclerosis, and various types of cancer^19,61,81,82^. There is ample evidence suggesting that the growth of cancer cells can be hindered by flavonoids^83,84^. Numerous advantageous properties of flavonoid glycosides have been documented, including their roles as antioxidants^85^, and in particular, kaempferol glycosides has garnered significant interest due to its potential applications in cancer chemotherapy and its diverse pharmacological effects^86^. Epidemiological studies indicate that the intake of foods rich in kaempferol may lower the risk of developing certain cancers, such as liver, colon, and skin cancers^87^. Preceding investigations have revealed that the mechanism through which flavonoids exert their effects involves the arrest of the cell cycle, inhibition of heat-shock proteins, suppression of tyrosine kinases, reduction in p53 protein levels, binding to estrogen receptors, inhibition of Ras protein, and modulation of Ras protein expression^88,89^.

Furthermore, Chen et al.^90^ have provided evidence for the anti-metastatic properties of anthocyanin. Anthocyanin, which belongs to the flavonoid family, triggers the apoptosis pathway by activating Caspase 3, apoptosis induction factor (AIF), and endonuclease G (pro-apoptotic factor). Moreover, it hinders the growth and advancement of cancer cells by causing cell cycle arrest in G0/G1 and G2/M phases. This is achieved through the upregulation of p21WAF1 and P27KIP1 expression and the downregulation of cyclin A and B expression^4^. Violanin, along with other delphinidin derivatives of anthocyanins, exhibits relatively potent antioxidant effects in scavenging free radicals when compared to other anthocyanin compounds^91^. Consequently, the pigmentation has the capacity to display significant antioxidant characteristics as a result of several factors, including the level of delphinidin derivatives, the existence of ortho-dihydroxy groups in the benzene ring, and the creation of complexes between anthocyanins and flavonoids^47^. Hence, these assumptions reinforce the concept that the presence of flavonoids and anthocyanins in *V. odorata* flowers could plausibly contribute to their anticancer characteristics.

A more efficient and logical approach is essential for the development of innovative therapeutic strategies. In this context, computational methods exhibit promising potential in the drug discovery and development process by minimizing both costs and time^92^. In the present in silico investigation, the Cyclooxygenase-2 receptor (COX-2) and Vascular Endothelial Growth Factor-1 (VEGF-2) receptors were selected as target proteins. The stable RMSD graphs indicate good convergence and stable conformations throughout the simulation. The results from the RMSD analysis demonstrated that the Kaempferol 3-*O*-rutinoside-bound protein complex achieved a comparatively more stable conformation than the other systems examined. Such stability is vital for an effective enzyme inhibitor, as it signifies a consistent and prolonged inhibition of enzyme activity. The calculated average RMSF values indicated that the majority of residues exhibited minimal fluctuations while bound to kaempferol 3-*O*-rutinoside throughout the simulation period, suggesting stable conformations of the amino acids. The rigidity of the protein structure during the simulation in ligand-bound states can be inferred from the RMSF plots. Additionally, the average Rg values demonstrate that the kaempferol 3-O-rutinoside compound maintains a highly rigid structure in relation to the COX-2 and VEGFR-1 receptors. Furthermore, the SASA results indicate that the target proteins possessed a significant surface area accessible to the solvent when the kaempferol 3-*O*-rutinoside ligand was not bound to the receptor. Upon binding with the kaempferol 3-*O*-rutinoside ligand, the SASA value decreased compared to the unbound state. Ultimately, the findings from the SASA analysis, in conjunction with the observations from the RMSD, RMSF, and Rg calculations, confirm that the kaempferol 3-*O*-rutinoside complex remains stable within the catalytic domain binding site of the COX-2 and VEGFR-1 receptors. The results obtained from the MM/GBSA method indicated that compound kaempferol 3-*O*-rutinoside exhibits favorable interactions, with ΔG values of -60.78 kcal/mol for COX-2 and -49.76 kcal/mol for VEGFR-1. An analysis of the binding free energy components suggests that the predominant factor contributing to this synergistic effect is the van der Waals energy component. Furthermore, the breakdown of total energies into contributions from the active site residues of COX-2 and VEGFR-2 identified several critical amino acid residues. In COX-2, the key residues include Val57, Tyr84, Val85, Ser88, and Arg89, while in VEGFR-2, the significant residues are Glu75, Ile78, Leu79, Ile82, and Cys171. The findings from our research hold significant value in elucidating the molecular mechanisms underlying the activity variations among synthesized compounds targeting COX-2 and VEGFR-2, as well as in the development of more effective selective inhibitors.

Although the isolated flavonoids exhibited significant anti-inflammatory and cytotoxic activities in vitro, their potential for therapeutic application is closely linked to effective drug delivery. Natural flavonoids often suffer from limited aqueous solubility and poor oral bioavailability, which can hinder their systemic absorption and therapeutic efficacy^93^. Therefore, advanced drug delivery systems—such as liposomes, polymeric nanoparticles, and solid lipid nanoparticles—have been explored to enhance their pharmacokinetic properties^94^. Nanoformulations, in particular, can protect flavonoids from enzymatic degradation, improve solubility, and enable controlled release and site-specific targeting, especially to inflamed or cancerous tissues^95^. These strategies hold promise in translating in vitro bioactivities into clinical efficacy. Hence, further studies are warranted to formulate the isolated flavonoids into biocompatible and targeted delivery systems.

## Conclusion

This study presents the first comprehensive investigation of the phytochemical composition and bioactivity of *Viola odorata* flowers, revealing them as a rich source of polyphenols, especially flavonoid glycosides. The isolation and structural elucidation of three key flavonoids—kaempferol 3-*O*-rutinoside, luteolin 7-*O*-glucoside, and 5,7-dihydroxy-3,6-dimethoxyflavone—demonstrated significant anti-inflammatory and cytotoxic activities. Importantly, molecular dynamics simulations provided mechanistic insights into the interactions between these flavonoids and key inflammatory and cancer-related targets, reinforcing their therapeutic relevance. Kaempferol 3-*O*-rutinoside showed superior biological activity and stable protein interactions, suggesting its promise as a drug lead. The correlation between phytochemical composition and biological activity not only validates the traditional use of *V. odorata* in herbal medicine but also positions its floral constituents as promising candidates for drug discovery. For researchers and clinicians, these findings offer a foundation for further pharmacological investigations into *V. odorata*-derived compounds, particularly in the development of plant-based anti-inflammatory and anticancer agents. Future studies may explore in vivo efficacy, bioavailability, and synergistic effects with existing therapies, paving the way for translational applications in integrative medicine.

## Supplementary Information

Below is the link to the electronic supplementary material.

Supplementary Material 1

## Data Availability

The datasets generated during the current study are available upon request from the corresponding author, Amal M. El Feky, at ammelfeky@hotmail.com.
