# Supplementary material for: Anti-inflammatory and cytotoxic assessment of flavonoids isolated from Viola odorata flowers with computer-guided docking study
Source: Sci Rep. 2025 Sep 18;15:32602. doi: 10.1038/s41598-025-19442-4 (PMC12446468; doi:10.1038/s41598-025-19442-4)
Supplement: Supplementary file 1 — Supplementary Material 1 [file 41598_2025_19442_MOESM1_ESM.docx]

**Supplementary file**

**Anti-inflammatory and cytotoxic assessment of flavonoids isolated from *Viola odorata* flowers with Computer-guided docking study**

# Amal M. El-Feky^1^, Ahmed A. El-Rashedy ^2,3^


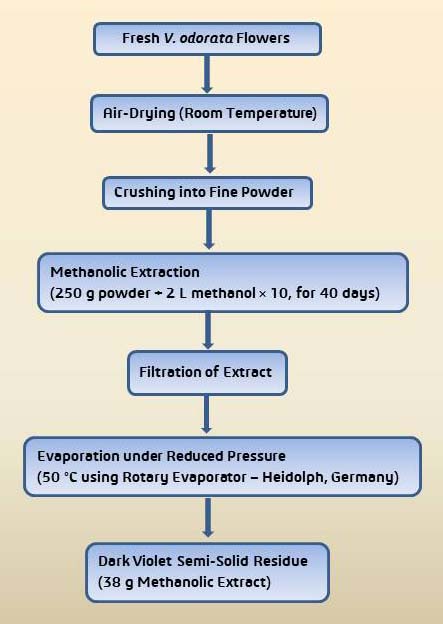


**Supplementary Figure S1.** Flow diagram illustrating the extraction steps of Viola odorata flower.


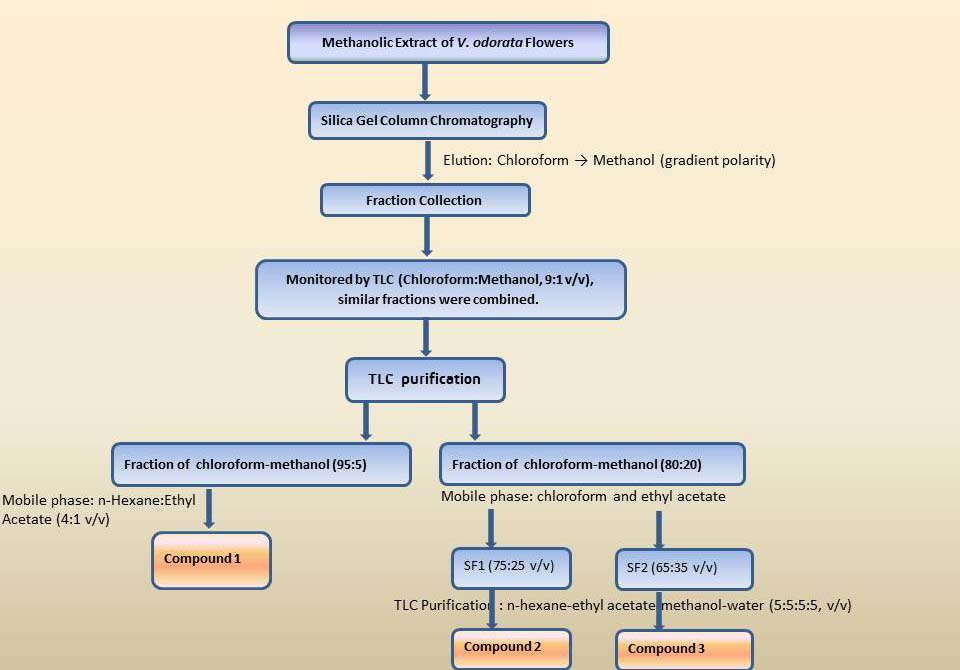


**Supplementary Figure S2.** Flow diagram illustrating the chromatographic separation, and identification of major flavonoids from Viola odorata flower methanolic extract.

**
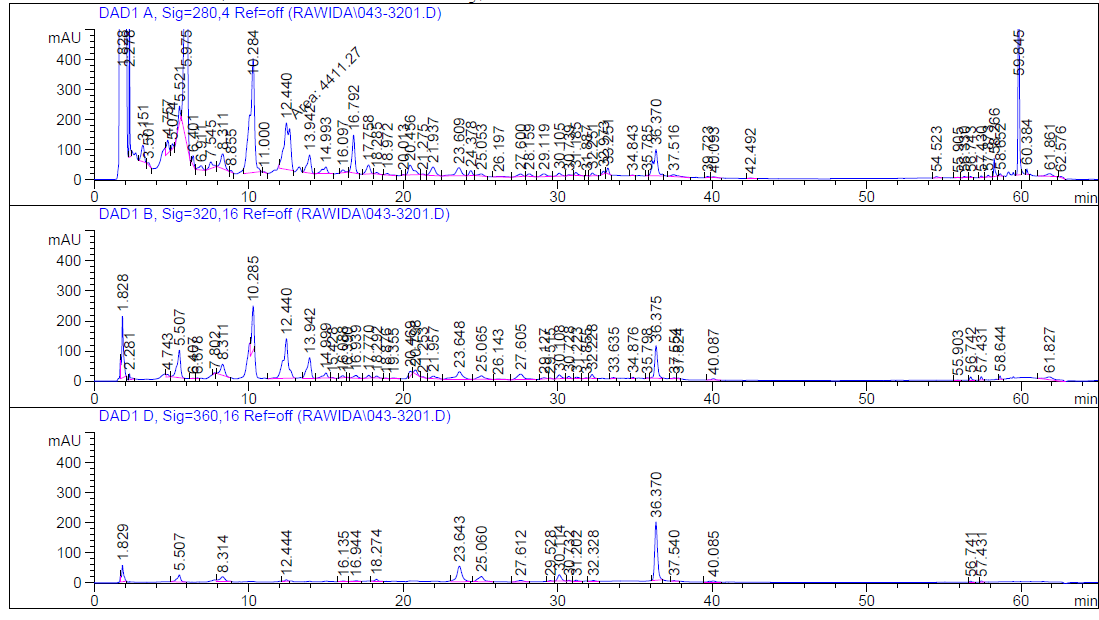
**

**Supplementary Figure S3.** HPLC chromatogram of phenolic acids and flavonoids in the methanolic extract of *V. odorata* flowers at 280,320, and 360 nm.

| 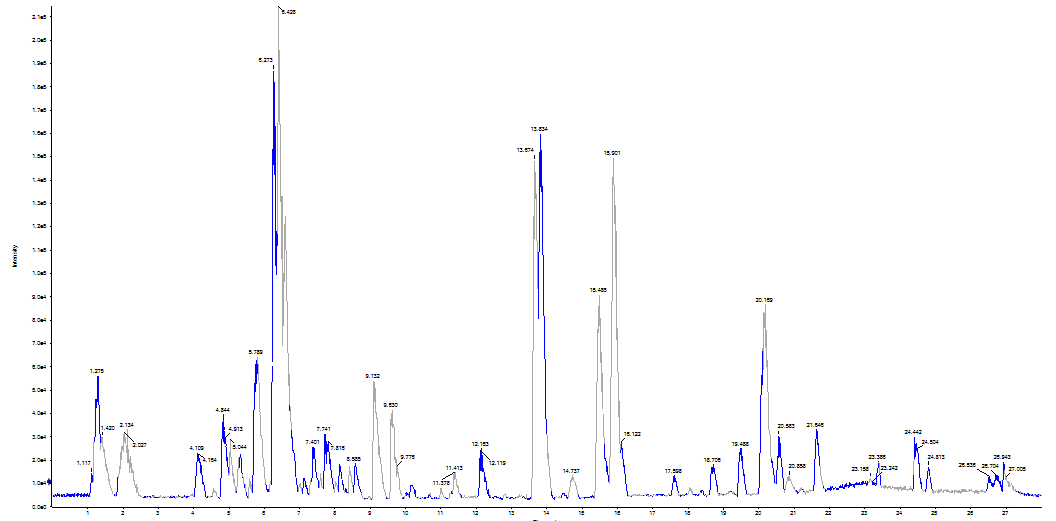  **A** |
| --- |
| 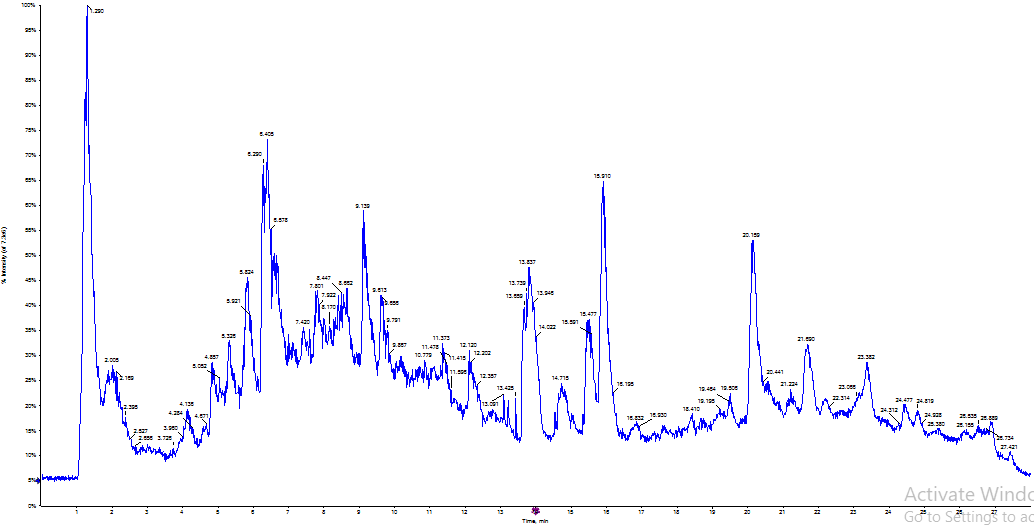  B |

**Supplementary Figure S4.** UPLC/ESI-MS chromatogram of the methanolic extract of *V. odorata* flowers in negative (A) and positive ionization mode (B).


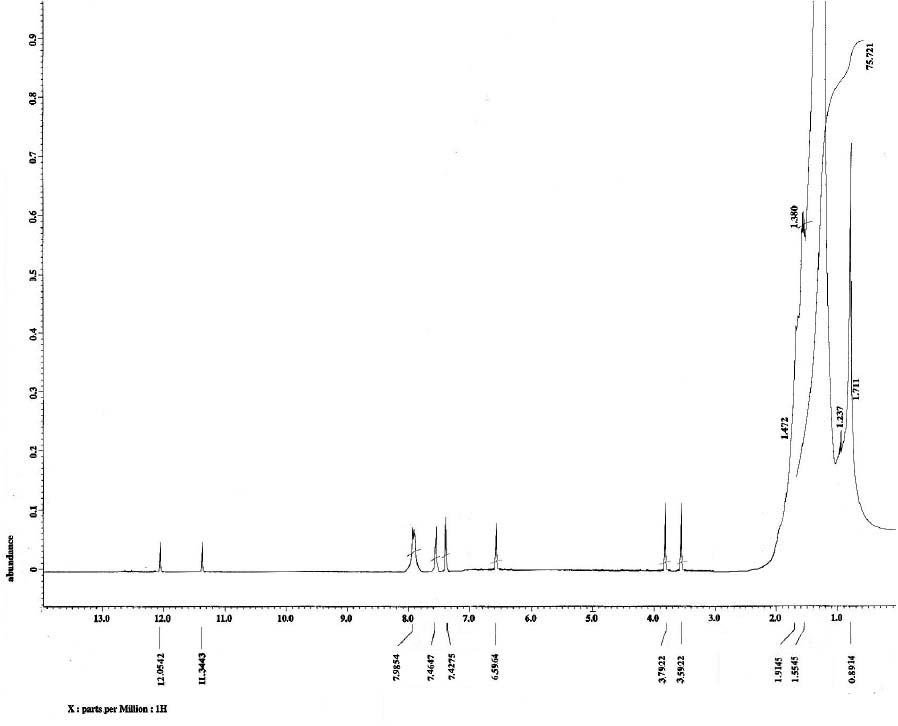


**Supplementary Figure S5.** ^1^H-NMR spectrum of compound 1.


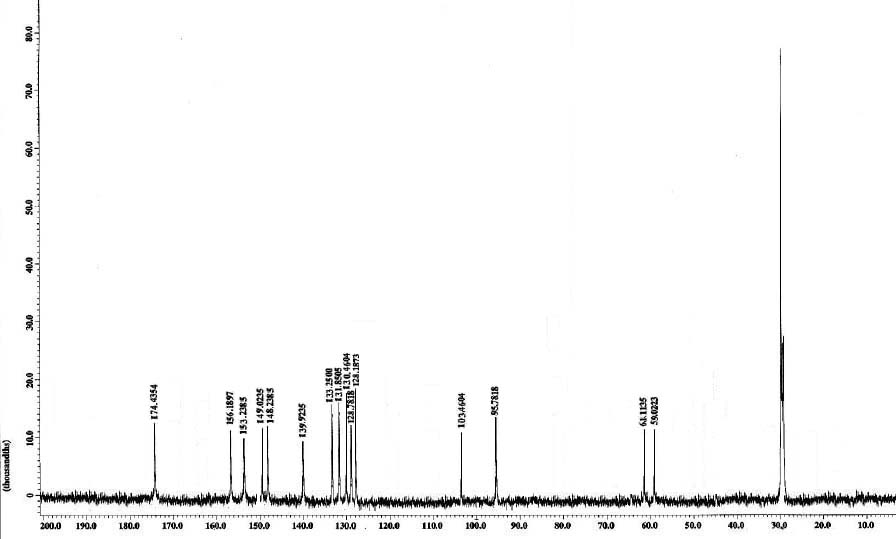


**Supplementary Figure S6.** ^13^C-NMR spectrum of compound 1.


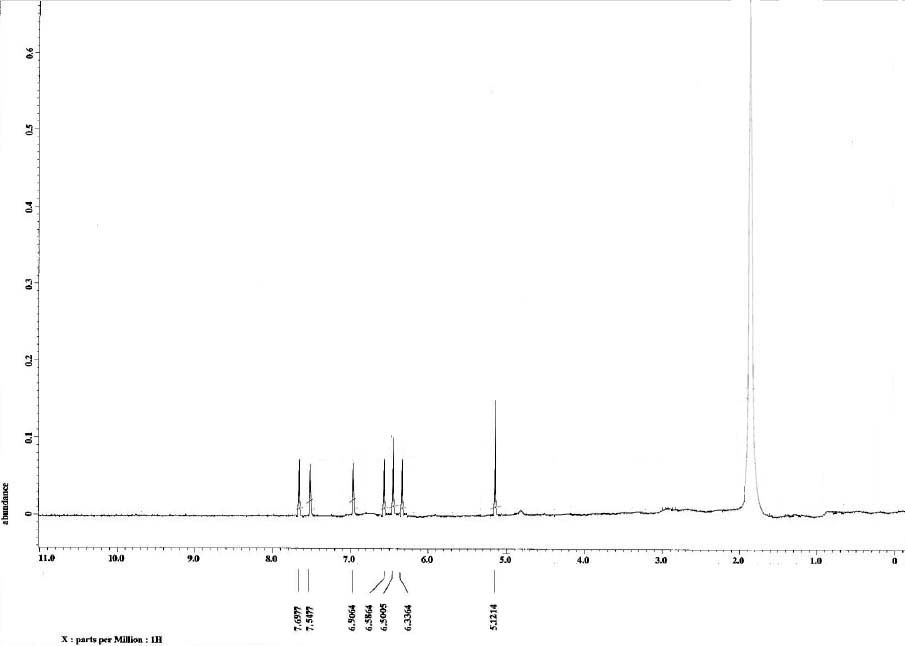


**Supplementary Figure S7.** ^1^H-NMR spectrum of compound 2.


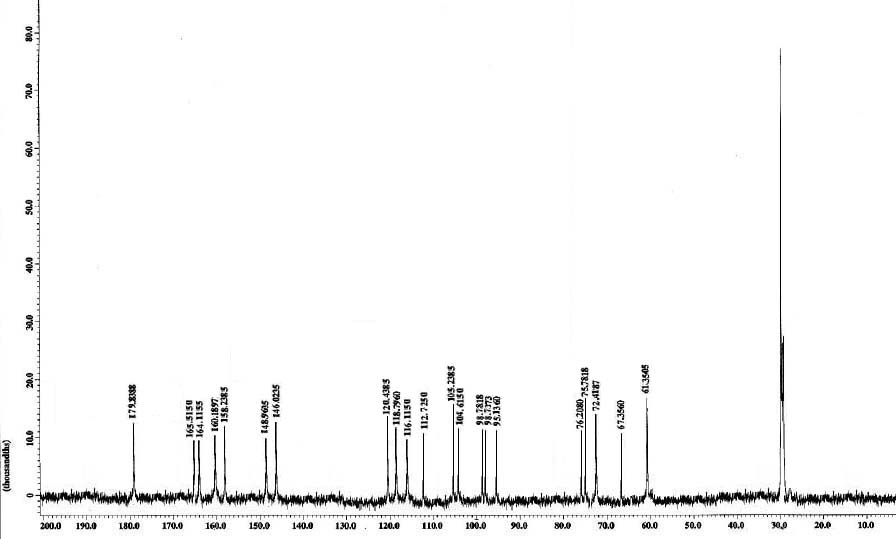


**Supplementary Figure S8.** ^13^C-NMR spectrum of compound 2.


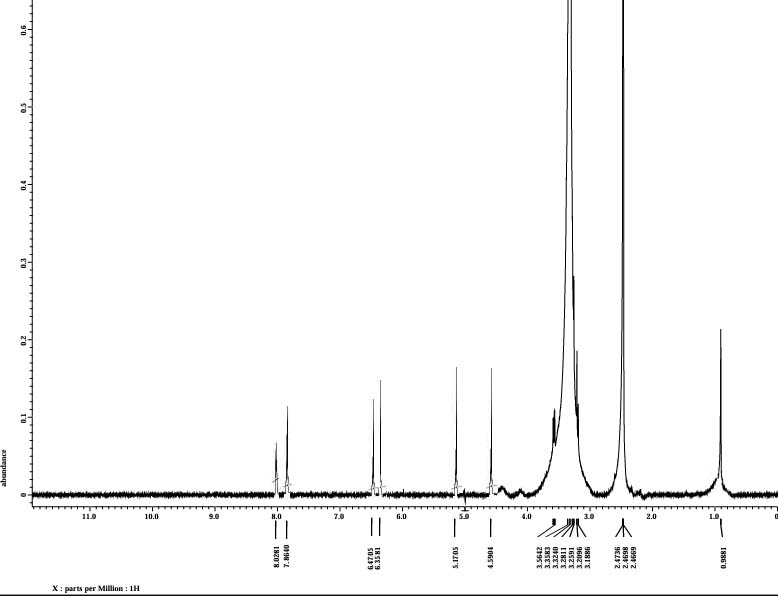


**Supplementary Figure S9.** ^1^H-NMR spectrum of compound 3.


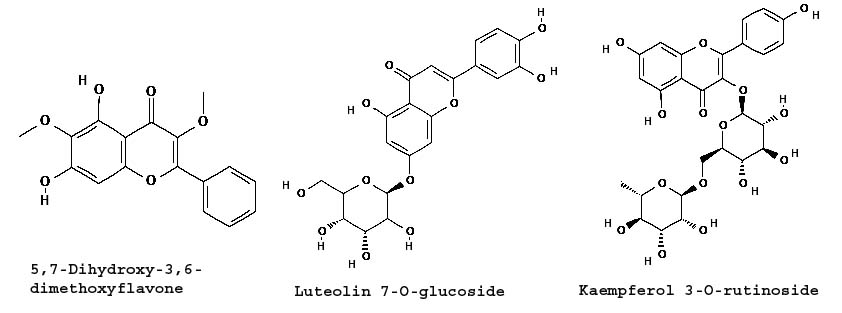


**Supplementary Figure S10.** Chemical structures of the major isolated flavonoids from *Viola odorata* flowers.
